# What Factors Dominate the Change of PM_2.5_ in the World from 2000 to 2019? A Study from Multi-Source Data

**DOI:** 10.3390/ijerph20032282

**Published:** 2023-01-27

**Authors:** Xiankang Xu, Kaifang Shi, Zhongyu Huang, Jingwei Shen

**Affiliations:** 1 Chongqing Jinfo Mountain Karst Ecosystem National Observation and Research Station, School of Geographical Sciences, Southwest University, Chongqing 400715, China; 2 Chongqing Engineering Research Center for Remote Sensing Big Data Application, School of Geographical Sciences, Southwest University, Chongqing 400715, China

**Keywords:** PM_2.5_, global trend analysis, multi-scale geographically weighted regression

## Abstract

As the threat to human life and health from fine particulate matter (PM_2.5_) increases globally, the life and health problems caused by environmental pollution are also of increasing concern. Understanding past trends in PM_2.5_ and exploring the drivers of PM_2.5_ are important tools for addressing the life-threatening health problems caused by PM_2.5_. In this study, we calculated the change in annual average global PM_2.5_ concentrations from 2000 to 2020 using the Theil–Sen median trend analysis method and reveal spatial and temporal trends in PM_2.5_ concentrations over twenty-one years. The qualitative and quantitative effects of different drivers on PM_2.5_ concentrations in 2020 were explored from natural and socioeconomic perspectives using a multi-scale geographically weighted regression model. The results show that there is significant spatial heterogeneity in trends in PM_2.5_ concentration, with significant decreases in PM_2.5_ concentrations mainly in developed regions, such as the United States, Canada, Japan and the European Union countries, and conversely, significant increases in PM_2.5_ in developing regions, such as Africa, the Middle East and India. In addition, in regions with more advanced science and technology and urban management, PM_2.5_ concentrations are more evenly influenced by various factors, with a more negative influence. In contrast, regions at the rapid development stage usually continue their economic development at the cost of the environment, and under a high intensity of human activity. Increased temperature is known as the most important factor for the increase in PM_2.5_ concentration, while an increase in NDVI can play an important role in the reduction in PM_2.5_ concentration. This suggests that countries can achieve good air quality goals by setting a reasonable development path.

## 1. Introduction

In the context of global change issues, air quality has received increasing attention from researchers around the world. Fine particulate matter (PM_2.5_) is particulate matter with an aerodynamic equivalent diameter less than or equal to 2.5 microns in ambient air, which has the characteristics of a long residence time in the atmosphere and long transmission distance, and has a great impact on human health and atmospheric environmental quality [1,2]. Many epidemiological studies have shown that many human health problems related to respiratory and lung diseases are associated with the concentration of PM [3,4]. In terms of the Global Burden of Disease (GBD), PM_2.5_ contributes to millions of premature deaths around the world [5,6]. Despite many countries and international organizations making many efforts to solve it, the threat of PM_2.5_ to the global environment and human health remains [7].

Numerous studies have been conducted regarding near-ground retrieval of PM_2.5_ concentration, and many achievements have been made. Currently, as the demand for PM_2.5_ research increases, the requirements for the high resolution and accuracy of near-surface PM_2.5_ concentration data increase. Currently, there are also many groups producing high-quality PM_2.5_ remote sensing products with different scopes and resolutions, thus proving highly convenient for application [8,9]. The Centers for Disease Control and Prevention has determined PM_2.5_ concentration for each year from 2003 to 2011, which is calculated at the country scale (https://wonder.cdc.gov/nasa-pm.html. Accessed on 16 January 2023); the United States Environmental Protection Agency has calculated daily PM_2.5_ concentration for the United States from 2002 to 2019, which contains spatial resolutions of 12 km^2^ and 36 km^2^ (https://www.epa.gov/hesc/rsig-related-downloadable-data-files. Accessed on 16 January 2023); Berkeley Earth provides real-time PM_2.5_ concentrations for the world at a spatial resolution of 0.1°, but it has only made the last two years of data available for download (https://berkeleyearth.org/archive/air-quality-real-time-map/. Accessed on 16 January 2023); Wei’s team has produced a 1 km PM_2.5_ concentration product for the whole of China, and has provided it on multiple time scales (https://zenodo.org/record/6398971. Accessed on January 16 January 2023); Van Donkelaar’s team has produced global annual PM_2.5_ concentrations from 1998 to 2021 and offers them at both 0.01° and 0.1° spatial resolutions; obviously, this is more suitable for conducting long time-series analysis on a global scale [10].

Studies on changes in PM_2.5_ and their driving forces have also been the focus of researchers’ attention [11,12]. Usually, PM_2.5_ concentrations are influenced by a combination of natural and anthropogenic factors [13]. Some studies have suggested that precipitation and green coverage rate of built-up areas cause the PM_2.5_ concentration to change [14,15], whereas others have indicated that the PM_2.5_ concentration has changed with growth in population and gross domestic product (GDP) [16,17,18]. However, the abovementioned studies focused on small-scale analyses and did not analyze long time-series data, so there are certain limitations in time and space; PM_2.5_ concentration changes are typically influenced by a variety of factors, therefore, natural factors such as precipitation, temperature, and vegetation, as well as socioeconomic factors such as GDP, population and urbanization rate should always be used for the analysis of the relationship with PM_2.5_ concentrations [19,20,21]. In addition, different analysis results may be obtained under different spatiotemporal scales. At present, there are relatively few studies on the factors influencing PM_2.5_ concentrations at the global scale, and these existing studies usually only analyze the impact factors of one aspect of PM_2.5_ or select a small number of factors for analysis [22]. Therefore, it is meaningful to study the multiple drivers of global PM_2.5_ concentrations.

The purpose of this study was to analyze the changing spatiotemporal trends in global PM_2.5_ concentration from 2000 to 2019 and to explore the driving factors, particularly for the years 2000, 2010 and 2019, using a multi-scale geographically weighted regression model (MGWR). Factors included changes in urbanization rate (UR), population density (PD), GDP per capita (GDP_per), total precipitation (TP), temperature at 2 m high (T2M), Normalized Difference Vegetation Index (NDVI), boundary layer height (BLH), wind speed (WS) and wind direction (WD). Using the multi-scale geographically weighted regression (MGWR) model to analyze the contribution of different influencing factors to PM_2.5_ concentration change, we can confirm the causes of air pollution in different regions. Identifying these influences can help find appropriate solutions.

## 2. Materials and Methods

### 2.1. Global Datasets

During this study, we used a variety of global datasets from 2000 to 2019 to analyze the spatial and temporal distribution characteristics and the impact factors of PM_2.5_. The impact factors were natural and socioeconomic. Datasets used included PM_2.5_ from the Atmospheric Composition Analysis Group of Washington University in St. Louis; NDVI product from Moderate-resolution Imaging Spectroradiometer (MODIS) on Aqua and Terra; surface pressure, total precipitation, 2 m temperature, boundary layer height and wind products from the fifth generation ECMWF reanalysis for the global climate and weather (ERA5); population density, urban ratio and GDP per capita statistical data from World Bank.

#### 2.1.1. Global Estimates PM_2.5_ Dataset

The global PM_2.5_ data generated by Van Donkelaar’s team were acquired from the Atmospheric Composition Analysis Group of Washington University in St. Louis (https://sites.wustl.edu/acag/datasets/surface-pm2-5/. Accessed on 16 January 2023). The dataset was computed using the GEOS-Chem chemical transport model and subsequently calibrated to global ground-based observations using geographically weighted regression (GWR) by combining Aerosol Optical Depth (AOD) retrievals from MODIS, MISR, and SeaWiFS instruments. The dataset, which has a span of 24 years (1998–2021), can provide a 0.01 × 0.01° and 0.1 × 0.1° spatial resolution on both the annual and monthly scale. Global PM_2.5_ product accuracy has also been verified using ground-based monitoring stations; the coefficient of determination performs well (R^2^ > 0.8), and the RMSE is also below 8.4 μg/m^3^ [23,24]. In this study, we used annual PM_2.5_ products with a 0.1 × 0.1° spatial resolution from 2000 to 2019.

#### 2.1.2. MODIS Vegetation Indices

MODIS on Terra and Aqua can capture information on vegetation, and the resulting global vegetation index product is provided by the 16-Day MODIS-Terra vegetation index from C6 (MOD13A2). The algorithm for this product chooses the best available pixel value from all the acquisitions from the 16 days. MOD13A2 has a spatial resolution of 0.01 × 0.01° and a time horizon of 2000 to the present. MOD13A2 includes two primary vegetation index layers, NDVI and EVI. In this study, we calculated the global annual NDVI using the Google Earth Engine (GEE) (https://developers.google.com/earth-engine/datasets/. Accessed on 16 January 2023). Specifically, the data for 2000 is an incomplete dataset for the year; this dataset starts in February, thus, the global average NDVI in 2000 was calculated from February to December.

#### 2.1.3. Meteorological Datasets

Changes in PM_2.5_ are sensitive to meteorological conditions, and meteorological data play a significant role in changes in PM_2.5_ concentrations [25]. The datasets of monthly averaged data on single levels from 1959 to the present produced by ERA5 were used in this study (https://cds.climate.copernicus.eu/. Accessed on 16 January 2023). Reanalysis combines model data with observations from across the world using the related laws of physics to complete a globally consistent dataset. These reanalysis datasets provide more than 260 meteorological variables, where the atmospheric variable spatial resolution is 0.25 × 0.25°, and ocean wave variable spatial resolution is 0.5 × 0.5°. Finally, total precipitation, 10 m u-component of wind, 10 m v-component of wind, boundary layer height and temperature at a height of 2 m were chosen for inclusion in the research.

#### 2.1.4. Nighttime Light Dataset

Nighttime light (NTL) satellite data can visualize the intensity of human activities at night. Because of its strong correlation with urban structure and socioeconomic characteristics, an increasing number of researchers are using NTL remote sensing data to study the economic development of cities [26,27]. The NTL dataset is commonly used by the Defence Meteorological Satellite Program Operational Linescan System (DMSP-OLS) and Suomi National Polar-Orbiting Partnership-Visible Infrared Imaging Radiometer Suite (NPP-VIIRS). The two NTL datasets differ in time span and quality assessment criteria, making it difficult to obtain perfect data for long time-series analysis. There are also some studies that extend NTL data for long time-series by integrating two datasets [28]. In this study, we use the 2000–2020 annual average NTL dataset (https://dataverse.harvard.edu/dataset.xhtml?persistentId=doi:10.7910/DVN/YGIVCD. Accessed on 16 January 2023) integrated by the autoencoder (AE) model using convolutional neural networks. The dataset has a spatial resolution of 0.01 × 0.01° and shows good agreement at both the pixel scale and city scale, with remote production validation with R^2^ values of 0.87 and 0.95, respectively [29].

#### 2.1.5. Global Statistical Data

Population density, urban ratio and GDP per capita data can reflect socioeconomic attributes and regional development levels. The World Bank provides a crucial tool to support key management decisions and key statistical information regarding the Bank’s operational activities in the context of the increasing demand for high-quality statistics. The World Bank database contains a large number of indicators covering statistics on various aspects of urban development, education, environment, economy, etc. Most of the data are sourced from its member countries, and therefore, the quality of the data is determined by the individual country systems. The population density, urban ratio and GDP per capita data used in this study are obtained from the World Bank database (https://data.worldbank.org.cn/. Accessed on 16 January 2023).

### 2.2. Methods

#### 2.2.1. Theil–Sen Median Trend Analysis and the Mann–Kendall Test

Theil–Sen median trend analysis is a robust trend calculation method which uses nonparametric statistics. The method is a linear regression through the median of the slopes of all lines at paired points. It is computationally efficient and insensitive to measurement errors and outlier data. In addition, it is often used for trend analysis of long time-series data [30], so it can objectively reflect spatiotemporal changes in PM_2.5_ concentrations.

(1) $$\mathrm{Kx}=Median\left(\frac{x_{j}-x_{i}}{j-i}\right)\left(2000\leq i<j\leq 2019\right)$$

where $K_{x}$ represents the slope of the fitted equation and $x_{j}$ and $x_{i}$ represent the value of the PM_2.5_ concentrations in years j and i, respectively. When $K_{x}>0$, the PM_2.5_ concentration shows an increasing trend. In contrast, when $K_{x}<0$, it shows a decreasing trend.

The Mann–Kendall (MK) test is a nonparametric statistical test used to determine the significance of trends in time series data [31,32]. The statistic Z of the trend test is calculated as follows:

(2) $$Z=\left\{ \begin{array}{l} \frac{S-1}{\sqrt{Var(S)}},\;S\;\text{>}\;0\\ 0,\;S=0\\ \frac{S+1}{\sqrt{Var(S)}},\;S\;\text{<}\;0 \end{array}\right.$$

S and $\sqrt{Var\left(S\right)}$ in equal 2 were calculated as follows:

(3) $$S=\sum_{j=1}^{n-1}\sum_{i=j+1}^{n}\mathrm{sgn}\left(x_{j}-x_{i}\right)$$

(4) $$\mathrm{sgn}\left(x_{j}-x_{i}\right)=\left\{ \begin{array}{l} \;1,x_{j}-x_{i}>0\\ \;0,x_{j}-x_{i}=0\\ -1,x_{j}-x_{i}<0 \end{array}\right.$$

(5) $$Var(S)=\frac{n(n-1)(2n+5)}{18}$$

where S is a statistical variable, $sgn\left(x_{j}-x_{i}\right)$ is the sign function, and Var(S) is the variance of S. Statistical Z is a bilateral trend test that can be combined with the normal distribution table to determine the significance level. In the study, statistic Z and the significance level correspond to Table 1.

#### 2.2.2. Multi-Scale Geographically Weighted Regression (MGWR)

According to the first law of geography, everything is spatially correlated with each other; specifically, the closer things are to each other, the greater the spatial correlation. Therefore, when regression analysis is performed on spatial data, its spatial location relationship should be considered. In order to explore the spatial nonstationarity of spatial data, the GWR model was proposed [33]. Distinct from global regression, the results calculated by the GWR model can be used to analyze the different relationships between the dependent and explanatory variables of the study object at different spatial scopes. Its essence is to obtain spatially continuous varying regression coefficients by weighting distances. The classical GWR considers spatial heterogeneity, but it uses a constant bandwidth in whole study area which can cause poor fitting results for some variables in local range.

The MGWR relaxes the assumption that all processes to be modeled are on the same spatial scale and overcomes the limitations of GWR very well. It captures the most appropriate bandwidth for each variable, so that the results of the regression obtained are more reliable [34]. The MGWR can be described as follows:

(6) $$Y_{i}=\beta_{bw0}(\mu_{i},v_{i})+\sum_{k=1}^{n}\beta_{bwk}(\mu_{i},v_{i})X_{ik}+\varepsilon_{i},i=1,2,3,\ldots ,n$$

where $Y_{i}$ is a dependent variable representing the PM_2.5_ concentration value at the spatial location $\left(u_{i},v_{i}\right)$, bw in $\beta_{bw0}\left(u_{i},v_{i}\right)$ and $\beta_{bwk}\left(u_{i},v_{i}\right)$ indicates the bandwidth for each variable, $\beta_{bw0}\left(u_{i},v_{i}\right)$ represents the intercept distance of the regression model, $\beta_{bwk}\left(u_{i},v_{i}\right)$ and $X_{ik}$ represent the coefficient and value of the explanatory variable k, respectively, and $\varepsilon_{i}$ is the random error.

The core of the MGWR model is the spatial weight function, and the selection of this function is crucial to the correct estimation of the regression parameters. In this paper, a Gaussian function is used as the weight function, which can be described as follows:

(7) $$w_{ij}=\exp\left[-{\left(\frac{d_{ij}}{b}\right)}^{2}\right]$$

where $d_{ij}$ represents the distance between i and j, and b represents the bandwidth used to describe the relationship between distance and weight. According to the MGWR model, the relationship between PM_2.5_ and explanatory variables can be established to better explain the spatial distribution characteristics of PM_2.5_.

## 3. Results

### 3.1. Global Spatiotemporal Patterns of PM_2.5_ in 2000–2019

**Figure 1:**
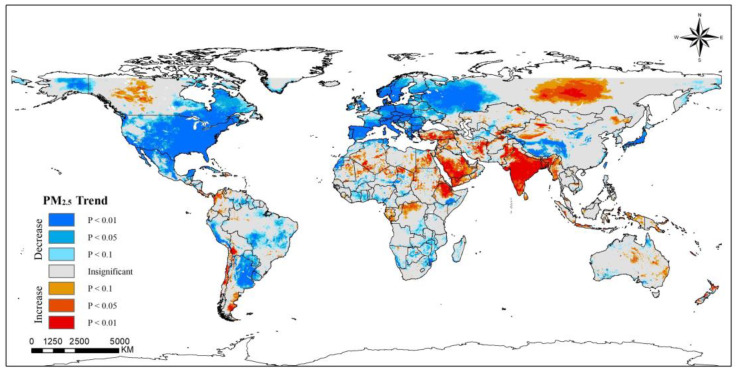
Spatiotemporal trends of PM_2.5_ concentrations worldwide (2000–2019).

The global spatiotemporal trends in PM_2.5_ concentration over the past twenty years were calculated, and the significance was tested (Figure 1). In most parts of the world, PM_2.5_ concentration showed a decreasing trend. The United States, eastern Canada, Japan, western Russia, northeast Argentina, the Tibetan Plateau region of China and a great number of countries in Europe show an extremely significant decreasing trend (*p* < 0.01), of which most are developed countries. Regions of western and eastern Africa (e.g., Namibia, Zambia, Nigeria, etc.), Turkmenistan, China’s coastal region and the Korean Peninsula are either generally significant or slightly significant (*p* < 0.05 and *p* < 0.1). In contrast, some countries or regions show increasing PM_2.5_ concentrations. Most regions of the Middle East area and India showed extremely significant increases (*p* < 0.01). Countries or regions such as central Canada, eastern Russia, Australia, Chile, and southern Argentina show different degrees of increase in the concentration of PM_2.5_ (*p* < 0.05 and *p* < 0.1). It could be seen that the PM_2.5_ concentration in developed countries has been declining more significantly than in other regions over the past two decades.

**Figure 2:**
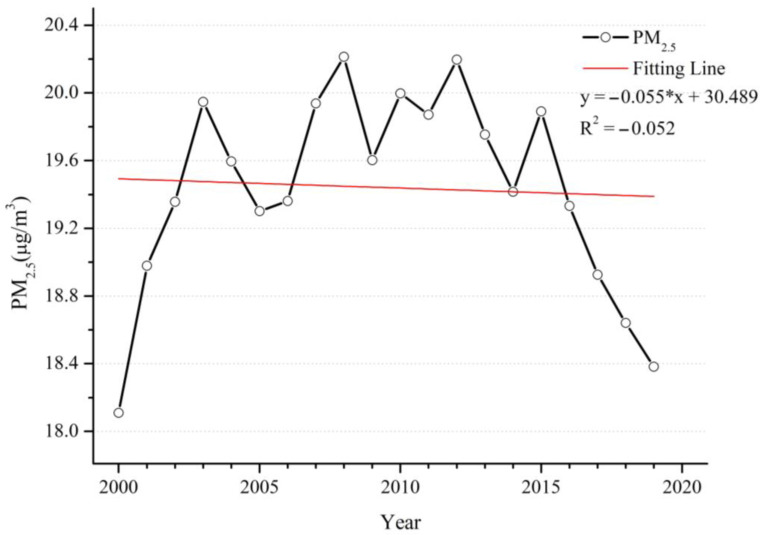
Time series of annual mean PM_2.5_ concentrations from 2000 to 2019.

During the twenty years, overall global PM_2.5_ concentration showed a nonmonotonic trend (Figure 2). It showed an increased from 2000 to 2003 then highly fluctuated over the next 12 years; during this time, the global economy was in a rapid development phase. However, in the past five years, it has begun to show a remarkable decreasing trend; this decreasing trend may be because the world has come to realize that environmental pollution should also be taken seriously as the economy develops. The decrease in PM_2.5_ concentration is significantly influenced by stringent policies adopted [35,36,37]. The fitting line of the time series shows that the trend line has decreased slowly, with a drop rate of −0.055 μg/m^3^/yr over the past 20 years. The highest annual average PM_2.5_ concentration appeared in 2008, with concentrations above 20.2 μg/m^3^. The WHO issued air quality guidelines in 2021, which set the annual average PM_2.5_ concentration at 5 μg/m^3^, and noted that when the concentration is more than 35 μg/m^3^, human health will be seriously threatened. At present, it seems that PM_2.5_ concentrations are moving closer to the WHO standard, but there is still a long way to go.

### 3.2. Spatial-Temporal Patterns of Natural Driving Factors

**Figure 3:**
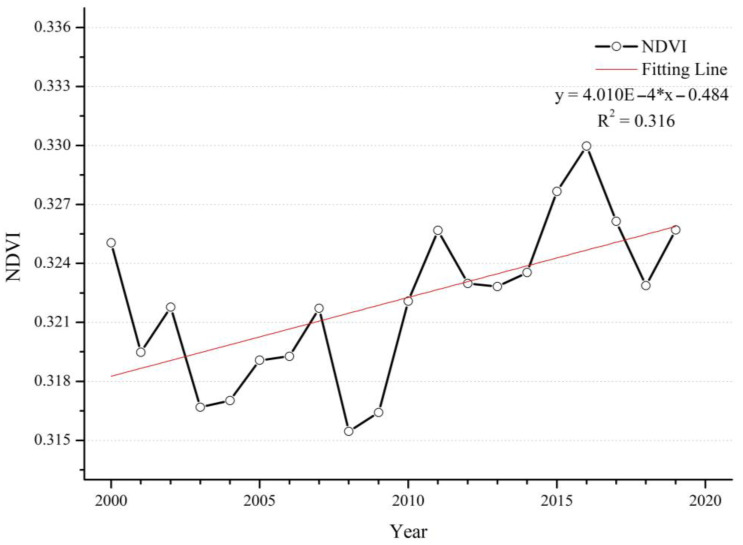
Time series of the annual mean NDVI from 2000 to 2019.

**Figure 4:**
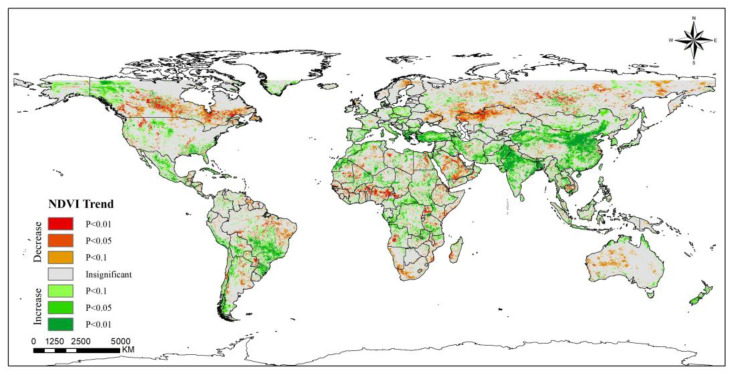
Spatiotemporal trends of NDVI worldwide (2000–2019).

NDVI is often used to reflect the state of terrestrial vegetation. There are a large number of studies showing that green spaces such as urban forests and parks effectively remove particle pollutants [38,39]. From 2000 to 2019 (Figure 3), the overall global NDVI showed an increasing trend. Before 2008, there was a slow decline, with an annual average decrease in NDVI of 0.01 over the 8 years, after which the trend of NDVI increased. The folding line of the global annual average value of NDVI was similar to a U-type; NDVI and PM_2.5_ showed opposite trends over the 20 years. Correspondingly, the highest value of PM_2.5_ and the lowest value of NDVI occurred in the same year. Regarding spatial distribution, most regions showed a trend towards becoming greener (Figure 4). The NDVI shows an extremely significant increase (*p* < 0.01) in central and eastern China, northwestern India, Turkey, Greece, southern Brazil, and southern Chile. The United States, Mexico and many countries in central Africa are also experiencing significant increases (*p* < 0.05). In contrast, many regions in the Middle East, South Africa, Nigeria, Canada and Australia observed various significant decreasing trends. It is worth noting that the relationship between NDVI and PM_2.5_ concentrations in some regions is opposite to that of the overall relationship. However, NDVI does not distinguish between agricultural land and other green spaces; at the same time, deforestation and agricultural expansion are occurring in many regions [40], such as India, where and the increasing trend is mainly contributed to by cropland [41]. For Canada, Guinea, Nigeria and Benin, although the NDVI showed a downwards trend, the PM_2.5_ concentrations in these regions also showed a downwards trend. This suggests that the variation in PM_2.5_ concentrations in each region may not be well explained from the perspective of trend changes in NDVI alone, and that such results may be dominated by changes in other factors.

**Figure 5:**
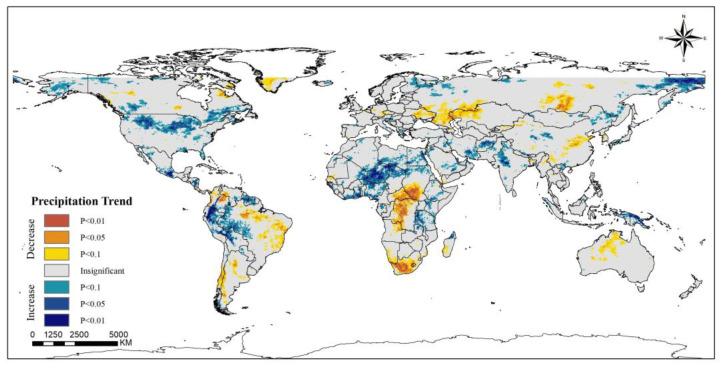
Global spatiotemporal trends of precipitation (2000–2019).

**Figure 6:**
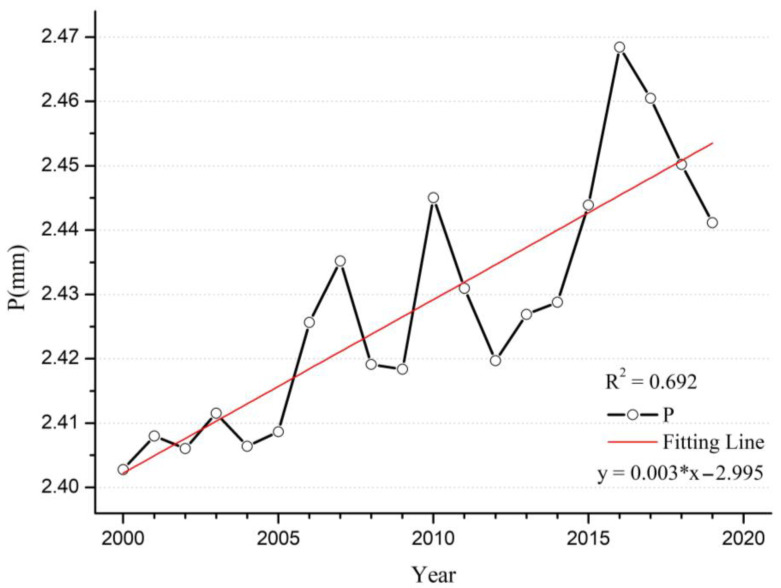
Time series of annual mean precipitation from 2000 to 2019.

Wet deposition is one of the most efficient ways to remove pollutants from the atmosphere [42], and precipitation is the most common type of wet deposition method. During this twenty-year period, more than half of the areas did not show a significant increase or decrease in precipitation. However, in other areas, there was a large spatially stratified heterogeneity in the trends of precipitation across the globe (Figure 5). Precipitation in the northern and eastern regions of the U.S. and Alaska showed increasing trends of varying significance, and the corresponding regional PM_2.5_ concentrations were on the decline, suggesting an important local correlation between precipitation and PM_2.5_ concentration. A similar situation occurred in Canada, Finland, Vietnam, and parts of western Africa. However, many areas, such as India, Saudi Arabia and Indonesia, showed a trend of increasing precipitation, but also an increasing trend in PM_2.5_ concentration; the increase in precipitation did not seem to have a visual impact on the decrease in PM_2.5_ concentration from Figure 1 and Figure 5. The main areas of reduced precipitation were Australia, eastern China, eastern Russia, northwestern Kazakhstan, central Africa, South Africa, and eastern and southern South America. The changes in PM_2.5_ concentration in these areas with reduced precipitation also show different trends. The time-series line chart shows a slowly fluctuating increase in global annual mean precipitation with a growth rate of 0.0025 mm/yr (Figure 6). There may be an important link between the increase in precipitation and global warming, but the increasing trend in precipitation may allow wet deposition to play a better role.

**Figure 7:**
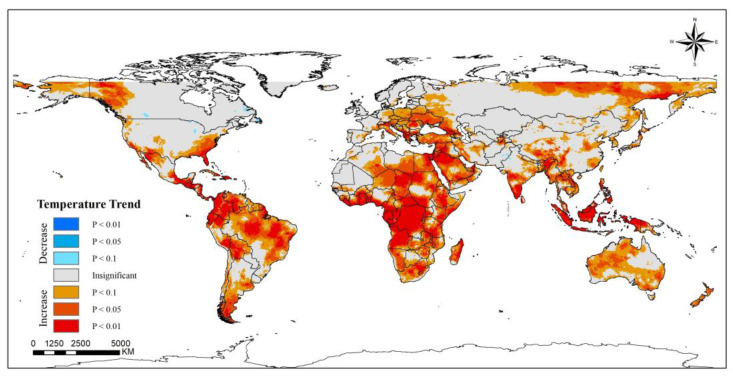
Spatiotemporal trends of temperature at 2 m high worldwide (2000–2019).

**Figure 8:**
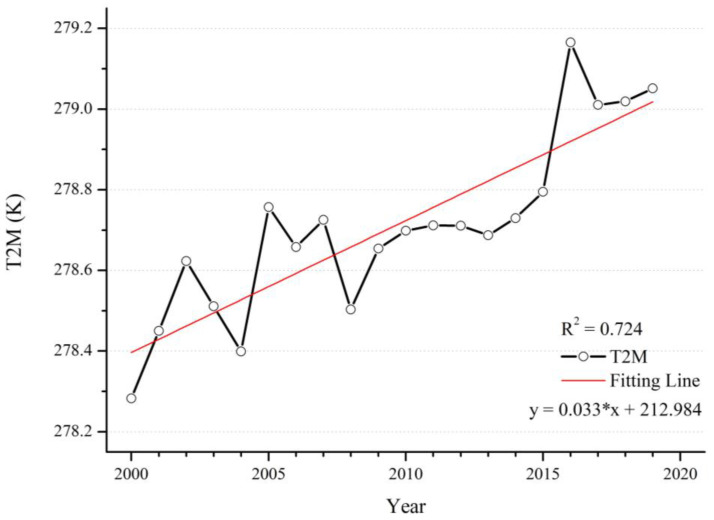
Time series of the annual mean temperature at 2 m high from 2000 to 2019.

Global warming is becoming an increasingly prominent issue, and has significant implications for the Earth’s natural environment. The generation of the greenhouse effect has led to changes in the characteristics of meteorological factors, resulting in changes in natural conditions such as regional precipitation and temperature, and it also has had an impact on the growth of terrestrial vegetation [43,44]. Near-surface temperature is an important component of climate conditions, and there is an inextricable relationship between PM_2.5_ concentration and temperature [45]. From 2000 to 2019, almost all regions showed a significant increase in temperature on a global scale, and even if there are very few regions, such as parts of Canada, the United States and Pakistan, with a decreasing trend, the overall temperature increase cannot be reversed (Figure 7 and Figure 8). In the 20 years, the temperature rose 0.88 K. The effect of this on PM_2.5_ is complicated because an increase in temperature has different effects on the different components of the air [46]. Therefore there is variability in the relationship between the warming effect and the change in PM_2.5_ concentration in different regions.

### 3.3. Spatial-Temporal Patterns of Socioeconomic Driving Factors

**Figure 9:**
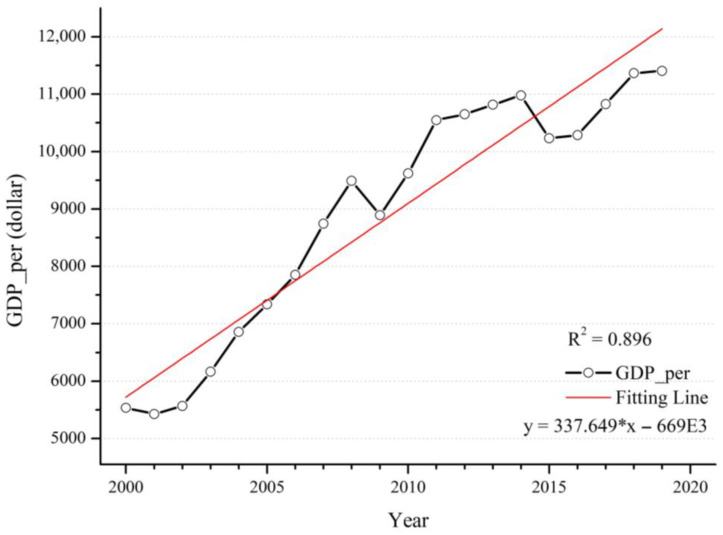
Time series of GDP per capita from 2000 to 2019.

Over the past two decades, the world economy has experienced a period of rapid development. Global GDP per capita showed a steady upwards trend, with a decline only in 2001, 2008 and 2015 (Figure 9). In 2001, over-saturated markets due to rapid technological development caused an economic crisis; in 2008, the United States triggered the subprime mortgage crisis, resulting in a hard hit to the world economy; in 2015, world industrial production experienced low growth, trade continued to slump, and financial market turmoil intensified. These three separate years of global events ultimately led to the economic downturn. Economic development and environmental protection are two inseparable topics. If the protection of the environment is neglected during economic development, the unrestricted emission of various pollution sources may lead to the increasing concentration of PM_2.5_, resulting in significant risks to human health.

**Figure 10:**
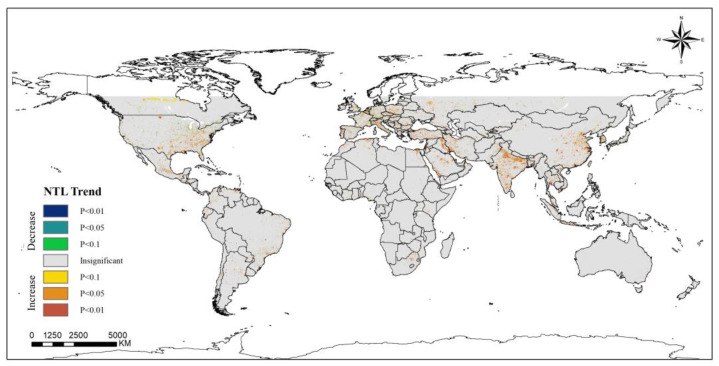
Spatiotemporal trends of NTL throughout the world (2000–2019).

NTL can be a good measure of the socioeconomic development of a region. Most developed countries, such as the United States, South Korea, and the European Union region, have shown an extremely significant increasing trend of NTL during the past 20 years, but the trend of PM_2.5_ concentration in these regions has not increased with the increase in human activity intensity. Instead, these areas show a decreasing trend, which is most likely because developed countries have invested more money in environmental management and protection and have achieved certain management results. In addition, different countries and regions have different development routes and priorities, resulting in economic development while failing to consider environmental pollution control. For this reason there is an increasing trend of PM_2.5_ concentration, mainly occurring in India, the Middle East, Chile, Colombia, and other developing regions. Moreover, in Canada, Japan and other regions, NTL shows a decreasing trend, and the PM_2.5_ concentration in these regions also shows the same trend; perhaps the reduction in the intensity of human activity has also effectively controlled the reduction in anthropogenic emissions (Figure 10).

**Figure 11:**
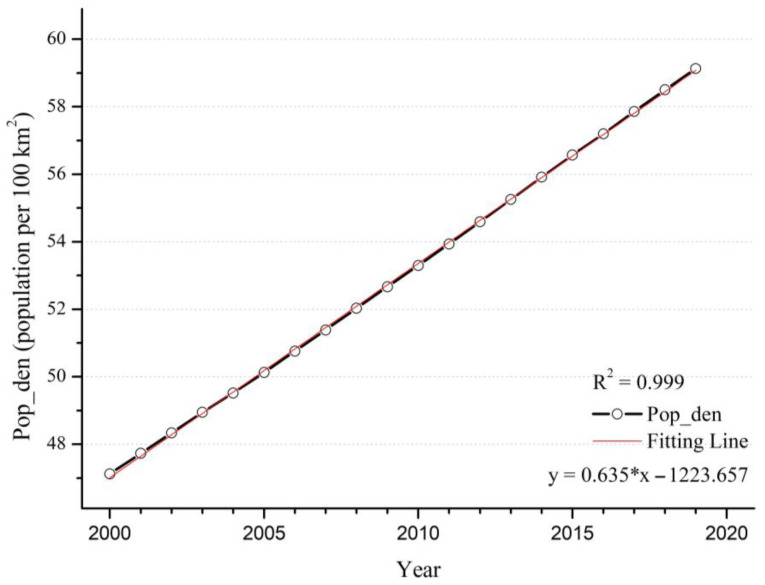
Time series of global mean population per 100 km^2^ from 2000 to 2019.

**Figure 12:**
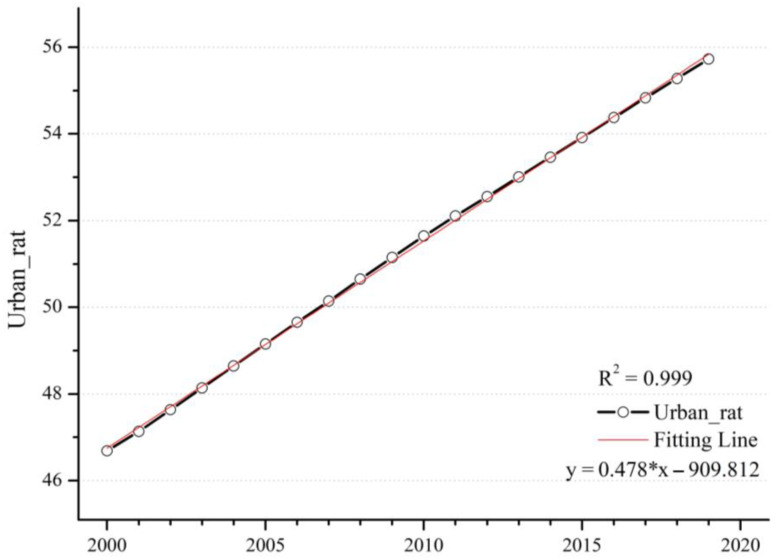
Time series of the global mean urban ratio from 2000 to 2019.

Changes in NTL and GDP are closely related to population density and urban ratio. The urban ratio is defined as the ratio of the urban population in the region to the total population in the region. As the urban ratio continues to increase, there is a large influx of rural population into urban areas, which eventually leads to a trend of increasing urban population density and decreasing population density in the periphery. The change in population density has an important impact on the intensity of human activities in the region, and it will also have a prominent effect on the PM_2.5_ concentration. Through Figure 11 and Figure 12, the trends of population density and urban ratio show a high degree of consistency over the 20-year period on the global scale. Within the first decade, the increase in population density and urban ratio may have contributed significantly to the increase in PM_2.5_ concentration, whereas within the second decade, PM_2.5_ concentration did not change at the same time. The decline in PM_2.5_ concentration indicates that the effect of increasing population density and urban expansion on the change in PM_2.5_ concentration is gradually weakening.

## 4. Discussion

### 4.1. Comparison with Traditional Linear Models

In this study, a MGWR model was applied to better understand the influence of natural and socio-economic factors on the spatial and temporal distribution of PM_2.5_ at national scales worldwide. Apart from the MGWR model, we compared different traditional linear models, such as ordinary least squares (OLS) and classic GWR. Table 2 shows the results of the three models. The traditional linear regression model ignores the spatial heterogeneity and the accuracy of the calculation was the worst. Variance inflation factor (VIF) was also calculated and it was found that the VIF values of the selected factors were all less than three. It is considered that there was no covariance between each factor, therefore GWR and MGWR can be used for regression. The GWR model takes into account the spatial heterogeneity of the study area, and the accuracy of the regression was also improved, however, because GWR defaults to all explanatory variables having the same spatial scale in the calculation process, this leads to less credible regression results in local areas. So, our study finally adopted the MGWR model, which also significantly improved the accuracy of the regression results, in addition to the interpretability of the model.

### 4.2. PM_2.5_ Concentrations Driving Factor Analysis

**Figure 13:**
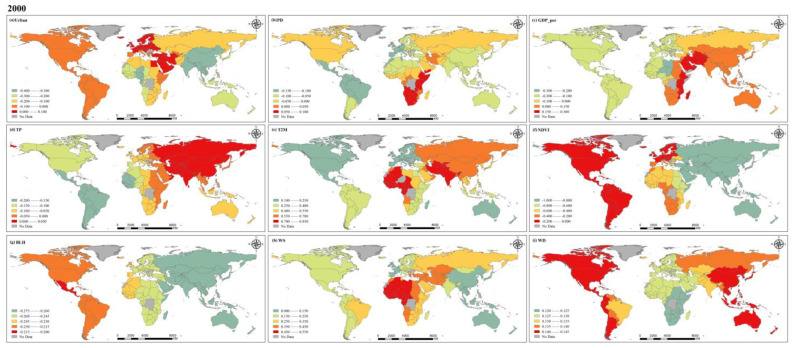
Spatial distribution of coefficients for factors in 2000.

**Figure 14:**
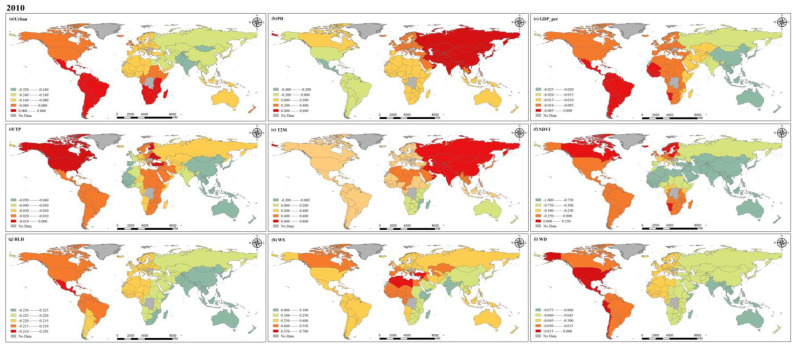
Spatial distribution of coefficients for factors in 2010.

**Figure 15:**
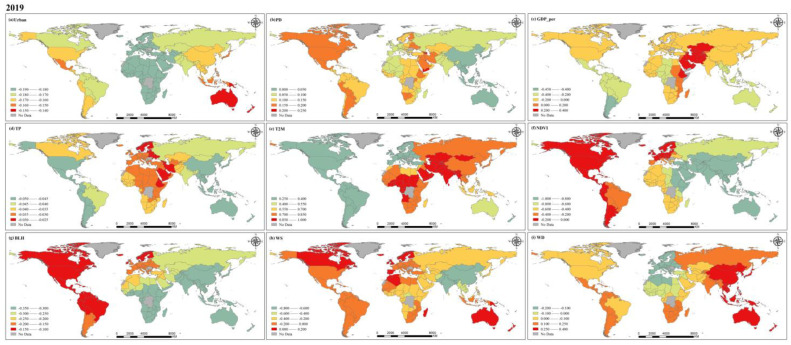
Spatial distribution of coefficients for factors in 2019.

Because of limited space, 2000, 2010 and 2019 are chosen here for in-depth discussion. Figure 13, Figure 14 and Figure 15 illustrate the regression coefficients of the factors we selected, and the figures are calculated based on the average of the selected factors across countries (Appendix A, Table A1, Table A2 and Table A3). As an important indicator of the degree of urbanization in a region, the urbanization rate can reflect the process and degree of population gathering in cities. Our results show that in 2000, the urbanization rate had a negative effect on PM_2.5_ concentration in most countries. In 2010, urbanization rates showed positive effects on PM_2.5_ concentrations in all regions except for some countries in South America and southern Africa, and in 2019, all countries maintained a negative effect on PM_2.5_. It can be assumed that with the increase in urbanization rate, the spatial layout within each country is more rational, which makes the production and living of people and the centralized management of the environment more efficient and orderly [47,48]. According to the urbanization rate statistics provided by the World Bank, countries in North and South America have relatively high urbanization rates globally, and they are already in a relatively mature state of urban development, maintaining a stable level of impact on PM_2.5_ compared to other factors. For countries in the Asian region, the negative effect of urbanization rate on PM_2.5_ concentrations mostly diminished over time, probably as a result of the increased influence of other factors on PM_2.5_ concentrations during the development process. In Europe, the opposite trend to Asia was shown, with urbanization rate playing an increasingly important role in the change of PM_2.5_ concentrations. Africa is relatively less developed than other regions and has relatively uneven development between countries, leading to greater heterogeneity. Population density, which reflects the density of the population living in a certain area, has a very important impact on PM_2.5_ concentration [49]. Generally speaking, the higher the population density, the higher the intensity of human activity. Similarly, dense urban and industrial areas are often accompanied by high population densities, which will also lead to increased emission of air pollutants. We calculated that population density had a negative effect on PM_2.5_ concentrations in most countries in 2000, except for central and southern Africa and the Middle East. However, in 2010, the negative effect of this factor on PM_2.5_ concentrations weakened, and reversed in 2019. The year-on-year increase in population density is illustrated in Figure 11, and the high population density further leads to more anthropogenic emissions, which can provide a significant influence on the increase in PM_2.5_ concentration [50]. In 2000, the effect of GDP per capita on PM_2.5_ concentration had significant geographical differences, with negative effects in all countries in the European and American region and positive effects in countries in the Asian and African regions. Such positive effects were more pronounced in developing countries, while some economically developed countries in the United States, Canada, and Europe showed negative effects. However, in 2010, the effect of GDP per capita on PM_2.5_ in all countries of the world showed a negative effect, with regression coefficients ranging from −0.025 to 0, which also indicates that its effect is relatively small. Years before 2010, as the global average annual PM_2.5_ concentration was at a high level, various problems caused by the increase in environmental pollution has made countries around the world also began to pay attention to the monitoring and management of PM_2.5_, and air pollution prevention and control bills introduced by various countries one after another may be an important reason for this shift [51,52]. In 2019, the negative impact on PM_2.5_ concentrations increased in most countries around the world as investment in environmental protection increased, but a positive impact has developed again in the Middle East and in eastern Africa, which may also contribute to the 20-year trend of increasing PM_2.5_ concentrations in these regions.

The influence of natural factors on PM_2.5_ concentration cannot be ignored. In general, moisture in the air is adsorbed and collected by suspended PM_2.5_, which eventually falls to the ground in the form of precipitation, while the occurrence of precipitation also leads to an increase in moisture in the air, which has a good effect on the removal of PM_2.5_ from the atmosphere [53]. In 2000, our results also show spatial distribution characteristics that are consistent with the above conditions. Precipitation has a negative effect on PM_2.5_ concentrations in regions with abundant mean annual precipitation, and this effect is particularly evident in regions with sufficient annual precipitation such as the Americas, Australia, southern Asia, and western Africa. In contrast, regions with low precipitation, such as southern and eastern Africa and northern Asia, show different results. Although seasonal precipitation is high in some of these regions, this study is based on annual averages at the national scale, weakening its spatial heterogeneity and certain characteristics. In addition, the intensity and frequency of precipitation can affect PM_2.5_ concentration variability [54]. In 2010 and 2019, the regression coefficients for precipitation were all between −0.05 and 0, indicating that the effect of precipitation on PM_2.5_ concentration appears to be weakened relative to other factors. The process of temperature influence on PM_2.5_ concentration is complex and a positive correlation between them is usually considered, which is consistent with our calculations at the global scale and shows a trend towards a stronger positive effect in many countries, which may have some connection with the global warming trend. However, some studies have also shown that higher temperatures enhance air convection, which in turn dilutes and diffuses PM_2.5_ and reduces local PM_2.5_ concentrations [55,56]. His diffusion effect may not be significant at larger spatial scales. During the past two decades, the positive effect of temperature on PM_2.5_ concentration has become increasingly significant due to the effects of global warming. Among the many factors, the regression coefficient of temperature is in the higher range, which means that the effect of temperature on PM_2.5_ concentration is crucial. Vegetation cover in all countries of the world showed different degrees of negative effects on PM_2.5_ concentration, which is consistent with the results of most studies, and it can be inferred that good vegetation cover can effectively reduce atmospheric PM_2.5_ concentration and is a very effective factor in suppressing PM_2.5_ concentration [57]. At these three time points, the variation in the regression coefficients of NDVI is relatively small worldwide, but shows a clear gradient, with the largest negative effects in Asia and Australia, followed by Africa, while in the Americas and Europe such negative effects are relatively weak. Boundary layer height determines the volume available for diffusion and transport of pollutants in the atmosphere [58]; when the BLH is large, the PM_2.5_ concentration mixed in it will be relatively low. The results of our study show that BLH has a negative effect on PM_2.5_ globally, and the spatial distribution is stable over the past 20 years, with the value of the regression coefficient remaining between −0.35 and −0.1. The degree of negative influence also increases gradually from west to east. Wind is an important condition in the process of diffusion and transport of pollutants in the atmosphere [59]. Wind speed and wind direction together affect the diffusion rate and direction of PM_2.5_ concentration. In 2000 and 2010, the positive effect of wind speed on PM_2.5_ concentrations was the largest in Africa and smallest in Asia, and by 2019, the effect of wind speed shifted to negative globally, probably due to the acceleration between ocean currents and sea breezes in the context of global warming, which further enhanced the diffusion of PM_2.5_ in the atmosphere and made PM_2.5_ concentration decrease.

Both socio-economic factors and natural factors lead to change in global PM_2.5_ concentration, and due to inconsistent development stages and speed of countries around the world, there is a strong spatial heterogeneity. According to the calculation results for developed countries, Europe and the Americas have more advanced science and technology and more reasonable urban structural layout, so the regression coefficients of these factors are relatively balanced. Conversely, economically underdeveloped countries still have to continue their economic development at the cost of the environment in order to seek better development, and so PM_2.5_ is more significantly affected with a positive influence. Throughout all three years, the positive effect of temperature on PM_2.5_ concentration was the largest of all variables. Although temperature is one of the natural factors, combustion and emissions from human activities are decisive causes of the greenhouse effect. The negative effect of NDVI on PM_2.5_ concentration is the largest in all three years. NDVI is influenced not only by the natural growth of vegetation, but also by human activities. The increase in artificial green areas can effectively reduce PM_2.5_ concentration. This also proves that the optimization of urban structure layout, the development of high technology, and the increase in vegetation all have positive effects on the reduction in PM_2.5_ concentration. The decrease in global average annual PM_2.5_ concentration in the past decade with the joint efforts of countries around the world confirms that the formulation of reasonable air pollution prevention and control acts can effectively monitor and manage PM_2.5_.

## 5. Conclusions and Limitation

The Theil–Sen median trend and MGWR approach for analyzing the spatiotemporal distribution of PM_2.5_ concentrations and the contributions of driving factors have improved our understanding of PM_2.5_. Currently, PM_2.5_ concentrations show a decreasing trend in the more economically developed countries, while many relatively backwards developing countries show an increasing trend, especially in India and the Middle East. From the perspective of natural and socioeconomic factors, we can see that developed and developing countries show opposite results under the same driving factors and developed and developing countries are at distinct stages of urban development. Developed countries have largely completed the urbanization process, while developing countries are experiencing rapid urbanization. This has a lot to do with the higher economic, technological and management levels of developed countries, which have experienced the lesson of “pollute first, treat later” and have invested more in environmental protection, and transferred many heavy polluting enterprises to developing countries, resulting in two opposite trends. The trends of global average NDVI and global PM_2.5_ concentrations show “U-type” and inverse “U-type” distributions, respectively, suggesting that the increase in green areas has the power to explain reduced PM_2.5_ concentrations. In conclusion, the increase in population density, socioeconomic development and urban expansion in the context of global warming and increasing climate extremes tend to lead to an increase in PM_2.5_ concentration. However, through effective management and by setting a reasonable development path, for example, in the process of urban planning, reasonably arranging the spatial layout of different functional industries to facilitate centralized management of industries that emit air pollutants, and then increasing investment in high-tech development to accelerate the goal of conversion to clean energy, the goal of synergistic development of economic development and environmental protection can be achieved in the end.

In this study, a limited number of drivers were selected to analyze their relationship with PM_2.5_ concentration. Emission sources that can have a direct impact on PM_2.5_ concentrations were not considered, making the discussion of the detailed causes of PM_2.5_ concentration changes somewhat limited. In addition, although the MGWR model is better than the OLS model and the GWR model in terms of fitting accuracy, there are still some limitations. The MGWR model is also a type of linear model, and a nonlinear expression may yield better results under complex spatial distributions. Moreover, the explanation of a single factor is not completely reliable; the process of PM_2.5_ concentration change should be a very complex process, and the interaction between these influencing factors is not clear. In future studies, we may consider more comprehensive influencing factors and try more models to explain the relationship between influencing factors and PM_2.5_ concentrations, and undertake further exploration of the interaction between factors.

## Figures and Tables

**Table 1:** Relationship between statistic Z and significance level.

Z Value	*p* Value	Significance Level
2.58 < |Z|	*p* < 0.01	extremely significant
1.96 < |Z| ≤2.58	*p* < 0.05	generally significant
1.65 < |Z| ≤1.96	*p* < 0.1	slightly significant
|Z| ≤1.65	*p* > 0.1	insignificant

**Table 2:** Comparison of multiple models.

Models	R^2^	Adjusted R^2^	AIC	AICc	RSS
OLS	0.648	0.628	314.139	317.876	57.715
GWR	0.786	0.729	283.407	303.760	35.054
MGWR	0.853	0.805	238.586	267.371	24.707

## Data Availability

Data is contained within the article and Appendix A.

## Appendix A

**Table A1:** Year-wise mean value of all the parameters in 2000.

County	UR	PD	GDP_per	PM25	TP	T2M	NDVI	BLH	WS	WD
AFG	22.0780	31.8291	179.4266	33.8495	0.0006	285.5928	0.1100	602.1169	1.5467	241.9826
ALB	41.7410	112.7382	1126.6833	21.7811	0.0029	286.1560	0.4692	393.1855	4.2350	241.0956
DZA	59.9190	13.0334	1765.0271	32.1160	0.0001	296.3192	0.1169	759.3276	2.7035	145.9869
AGO	50.0870	13.1511	556.8362	20.2957	0.0029	295.0195	0.5204	629.8076	4.7150	85.6959
ARG	89.1420	13.4728	7708.0991	15.1521	0.0026	286.4868	0.3726	662.6028	4.1936	210.3534
ARM	64.6660	107.8187	622.7409	23.8468	0.0020	279.2242	0.3108	411.0739	2.5497	284.8812
AUS	84.2350	2.4931	21,697.7085	4.6080	0.0018	294.3353	0.3452	760.6279	1.6857	113.0352
AUT	60.2130	97.0158	24,625.6007	15.4217	0.0035	280.4046	0.5436	434.3101	4.4736	239.8334
AZE	51.3860	97.4348	655.1199	26.1463	0.0013	286.8007	0.2662	459.8327	2.7473	307.7411
BGD	23.5900	980.7011	418.0689	52.7968	0.0068	297.9745	0.4662	433.1246	4.2884	92.8961
BLR	69.9730	49.1994	1276.2880	16.7132	0.0020	281.0086	0.5070	570.2219	3.3204	232.1806
BEL	97.1290	338.5485	23,098.8865	13.3689	0.0029	283.8709	0.6302	606.3830	2.9343	251.8785
BLZ	45.3980	10.8422	3364.4917	18.3197	0.0038	297.7080	0.6944	530.6097	1.5121	240.5799
BEN	38.3330	60.8899	512.6739	48.8258	0.0028	300.5466	0.5026	646.1343	6.8092	75.5987
BTN	25.4180	14.8496	718.1963	20.8942	0.0085	279.4159	0.4793	281.6225	1.9581	99.0074
BOL	61.7870	7.7709	997.5818	25.1072	0.0039	292.7631	0.5652	562.5574	2.5150	158.8676
BIH	42.3840	73.2652	1484.1761	27.5371	0.0026	284.2731	0.5966	473.5262	4.7509	239.8284
BWA	53.2190	2.8997	3522.3108	17.8380	0.0019	294.1540	0.3554	720.8429	3.4470	98.1352
BRA	81.1920	20.9126	3749.9108	14.8498	0.0052	297.5663	0.6446	480.5716	1.4058	146.6187
BRN	71.1640	63.2194	18,012.5022	5.6315	0.0069	299.8415	0.6924	380.4360	0.8500	74.4657
BGR	68.8990	73.8513	1621.2430	26.0335	0.0013	284.9830	0.5071	506.0910	4.1326	246.6907
BFA	17.8440	42.4267	255.7187	40.7396	0.0016	301.4529	0.3537	682.2506	7.3906	75.8445
BDI	8.2460	248.3984	136.4640	31.9833	0.0038	293.5932	0.4995	586.9069	6.1900	87.9110
KHM	18.5860	68.8604	300.6137	17.3857	0.0056	300.1102	0.5829	495.5282	8.7732	70.0928
CMR	45.5420	32.8192	681.1020	46.4291	0.0050	297.1607	0.5602	492.6553	6.1173	88.5900
CAN	79.4780	3.4226	24,271.0021	3.6574	0.0016	267.0108	0.2358	401.5914	0.9749	217.8069
CAF	37.6390	5.8436	251.8328	32.3960	0.0036	298.4704	0.6555	560.6827	5.8547	86.8668
TCD	21.6370	6.6357	166.1757	50.6063	0.0007	299.4048	0.2114	681.5718	7.3860	75.8585
CHL	86.0730	20.6344	5100.2541	13.7580	0.0042	281.9560	0.3043	504.8170	6.2987	227.9254
CHN	35.8770	133.9719	959.3725	27.8467	0.0022	279.4784	0.2905	542.6256	1.5997	213.1305
COL	73.9570	35.7188	2520.4811	16.7692	0.0099	296.4643	0.6055	346.5086	0.5432	181.0906
COM	28.0800	291.4336	647.4258	10.6556	0.0024	298.1979	0.6449	585.0796	6.3745	91.0849
COG	58.6950	9.1579	1032.1376	34.2481	0.0045	297.2450	0.5938	392.7598	6.0708	88.7862
COD	35.1220	20.7785	405.2162	35.1962	0.0045	296.8253	0.6821	433.8509	5.9154	88.6850
CRI	59.0520	77.6022	3789.0539	14.9833	0.0089	295.8813	0.6398	415.7976	1.6097	238.0919
CIV	43.1550	51.7442	1007.4674	26.8967	0.0032	299.0755	0.5293	566.5360	6.2012	78.4459
HRV	53.4280	79.9195	4887.7137	20.0698	0.0026	285.7910	0.5890	474.9056	4.7876	239.8142
CUB	75.3230	103.5980	2747.1003	11.9491	0.0020	298.2920	0.6334	670.7790	0.4833	175.6806
CYP	68.6480	102.0874	14,388.3477	20.6622	0.0013	292.7031	0.3167	553.2762	1.1752	254.4217
CZE	73.9880	132.7173	6029.0382	19.3637	0.0022	282.3130	0.5611	547.9158	4.0450	238.6683
DNK	85.1000	125.8453	30,743.5477	2.2790	0.0010	253.7751	−0.0335	199.3058	0.8690	172.3289
DJI	76.5320	30.9567	768.1836	35.7283	0.0007	301.5547	0.1125	802.4154	7.1028	64.7858
DMA	65.2650	92.8667	4787.8014	12.5750	0.0020	298.7954	0.7040	787.4301	0.7327	40.9683
DOM	61.7530	175.3533	2869.1781	11.5998	0.0023	296.8789	0.6506	546.2463	1.6014	84.6368
ECU	60.2990	51.0594	1445.2793	13.7362	0.0087	292.7888	0.4890	316.9937	0.4371	249.6579
EGY	42.7970	69.1462	1450.4762	40.9264	0.0000	295.3019	0.1124	611.8229	4.5002	87.9643
SLV	58.9120	284.1665	2001.5400	28.1796	0.0036	297.7833	0.6780	594.0580	1.4049	226.3673
GNQ	49.0920	21.6107	1725.5576	31.2369	0.0089	296.6764	0.4818	356.0040	6.1448	94.8720
ERI	26.5870	22.6972	308.1342	34.0939	0.0008	299.9710	0.1626	690.7507	8.1488	67.6865
EST	69.3680	32.9555	4070.6090	9.0725	0.0021	280.1713	0.4808	561.5966	0.8786	88.1589
ETH	14.7400	66.2248	124.4608	19.0285	0.0026	296.0029	0.3899	768.6521	5.6664	83.9350
FJI	47.9080	44.3903	2069.3175	6.3497	0.0071	297.6915	0.7419	487.4509	6.9668	126.4357
FIN	82.1830	16.9940	24,345.9148	5.7739	0.0020	276.7162	0.4203	537.4464	0.8106	96.9632
FRA	75.8710	111.2421	22,419.6948	12.5566	0.0029	284.7556	0.6148	547.0696	4.0811	255.9357
GAB	78.8790	4.7672	4135.9924	25.5043	0.0057	297.2041	0.4620	398.9363	6.0869	88.3177
GEO	52.6380	71.3309	749.9085	19.6521	0.0030	280.2393	0.4869	321.5960	2.7419	268.9658
DEU	74.9650	235.5968	23,694.7605	14.4042	0.0025	283.1206	0.6122	578.5321	3.0288	235.6645
GHA	43.9290	84.7273	258.4710	38.9228	0.0027	300.2450	0.4944	595.5582	6.3969	78.3945
GRC	72.7160	83.8309	12,072.9294	18.9479	0.0015	287.4909	0.4884	471.7066	3.4461	241.5302
GTM	45.3320	108.1538	1664.2990	29.2672	0.0049	295.3244	0.6869	458.7450	1.5379	222.1088
GIN	30.8690	33.5371	363.4823	31.1368	0.0048	298.8392	0.5809	554.7373	6.7179	76.1871
GNB	36.2430	42.7207	308.9103	39.1327	0.0033	300.8018	0.5674	510.8033	6.6601	78.3687
GUY	28.6940	3.7933	954.4003	13.7076	0.0055	297.9446	0.6759	472.8967	0.4275	149.9103
HTI	35.6000	307.1046	805.0256	12.3057	0.0019	298.0041	0.5760	515.2011	2.1137	88.6423
HND	45.4580	58.7587	1093.1081	25.1281	0.0040	295.9077	0.6654	511.7707	1.5186	245.6845
HUN	64.5750	113.9363	4624.2817	22.0857	0.0014	285.0736	0.4931	538.7070	4.7223	238.8301
ISL	92.4010	2.8050	32,096.3723	4.5094	0.0034	274.5433	0.1527	508.5296	0.5950	189.5988
IND	27.6670	355.3677	443.3142	34.9695	0.0032	296.5155	0.3923	581.7520	1.8893	162.2188
IDN	42.0020	116.7572	780.1902	10.8315	0.0092	297.9742	0.6675	325.5052	1.3315	95.2109
IRN	64.0420	40.2904	1670.0097	39.8337	0.0007	291.2217	0.1177	707.7281	4.2147	290.1329
IRQ	68.4960	53.7247	2058.2644	47.3930	0.0005	295.9818	0.1214	714.0609	1.9945	204.3714
IRL	59.1550	55.2355	26,334.5672	8.8693	0.0034	282.8344	0.6870	720.5282	1.8207	318.4994
ISR	91.2030	290.6192	21,061.4823	23.1614	0.0008	292.8159	0.2026	507.2279	1.1623	67.9278
ITA	67.2220	193.6082	20,137.5912	18.0839	0.0029	285.9755	0.5266	414.2281	4.5311	242.0215
JAM	51.8140	245.1245	3392.1239	16.2660	0.0028	298.2675	0.7028	528.4640	0.4771	124.8375
JPN	78.6490	347.9918	39,169.3596	12.5862	0.0047	284.6239	0.5852	518.8859	2.7491	230.1247
JOR	78.2700	58.0518	1651.6218	26.7614	0.0002	292.2210	0.1132	660.0089	1.2624	66.0555
KAZ	56.0980	5.5131	1229.0012	15.1920	0.0010	280.1412	0.1870	564.2142	1.0764	250.5042
KEN	19.8920	56.1629	397.4827	16.3608	0.0013	298.2487	0.3205	939.0473	5.7940	101.6190
KWT	99.0000	114.7656	18,440.3785	52.6699	0.0003	299.0618	0.0839	677.1914	3.4631	26.9840
KGZ	35.2980	25.5391	279.6196	23.1515	0.0027	274.2499	0.1863	292.7439	1.6498	250.5258
LAO	21.9770	23.0663	325.1869	22.9004	0.0056	295.7368	0.6422	402.0839	5.1345	74.4398
LVA	68.0670	38.0660	3361.6409	14.9601	0.0019	280.6965	0.5192	580.0885	0.8342	179.7964
LBN	86.0000	375.6377	4491.6419	27.0500	0.0018	290.4683	0.3016	454.8156	0.5243	268.4361
LSO	19.5480	66.9567	436.4881	23.6788	0.0034	284.1797	0.3842	529.9560	0.9590	106.4457
LBR	44.3310	29.5727	306.8339	19.7021	0.0058	297.7780	0.5444	423.0724	5.6095	80.5398
LBY	76.3870	3.0451	7142.7718	34.1456	0.0001	294.7351	0.1071	613.1045	3.7460	107.4708
LTU	66.9860	55.8318	3293.2300	15.8328	0.0020	281.2286	0.5123	590.3737	1.7673	220.0129
LUX	84.2160	179.5473	48,659.5989	12.5844	0.0031	282.4963	0.6448	563.4920	3.4993	247.1581
MKD	58.5480	79.6834	1861.8981	30.2412	0.0014	284.0713	0.4836	494.2013	4.2231	243.0377
MDG	27.1210	27.1122	293.6072	9.9970	0.0039	295.6260	0.4926	612.3831	4.4417	85.8211
MWI	14.6100	118.2515	156.3858	16.3842	0.0040	294.5150	0.4333	576.2796	5.3808	86.4732
MYS	61.9770	70.5958	4043.6629	10.6796	0.0080	298.5966	0.7057	329.2694	1.1416	164.3602
MLI	28.3560	8.9711	270.5430	42.4320	0.0006	301.5000	0.1922	740.7580	6.2072	84.1524
MLT	92.3680	1219.0219	10,432.3281	13.7500	0.0010	292.3575	0.2484	612.6875	3.1328	228.2837
MRT	38.0910	2.5519	676.5690	52.1978	0.0002	300.4292	0.1116	674.1965	4.9332	86.6109
MUS	42.6700	584.6665	3929.0755	14.5286	0.0019	296.7129	0.6466	850.4476	4.5999	89.5251
MEX	74.7220	50.8757	7157.8145	13.9014	0.0020	293.8660	0.4250	641.3558	1.1471	247.7002
MDA	44.5890	101.8137	440.6720	18.1592	0.0015	284.0422	0.4543	586.3882	4.4783	245.3239
MNG	57.1330	1.5432	474.2171	14.7267	0.0007	273.8502	0.1689	588.8943	1.0681	270.4900
MAR	53.3350	64.5164	1334.9435	21.9904	0.0007	291.2152	0.1744	628.4530	1.6099	227.1025
MOZ	29.0980	22.5234	319.3596	16.5914	0.0033	296.3921	0.5950	632.4653	4.5914	89.7289
MMR	27.0250	71.4871	146.6046	24.3684	0.0067	296.0873	0.5785	391.8436	4.1761	74.0680
NAM	32.3730	2.1798	2185.6041	13.1942	0.0009	294.0247	0.2344	731.0292	3.3325	96.6204
NPL	13.3970	167.0115	229.4904	32.7714	0.0058	284.5282	0.4414	326.2043	2.6105	129.7383
NLD	76.7950	471.7273	26,214.4986	14.8299	0.0027	283.9303	0.6037	632.3657	2.1224	245.3230
NZL	86.0210	14.6508	13,641.1027	6.8349	0.0048	283.4144	0.6530	556.9278	5.1602	283.4279
NIC	55.1850	42.1249	1007.4998	16.0619	0.0039	297.8682	0.5990	649.7882	1.5067	242.0101
NER	16.1860	8.9457	197.8327	60.7594	0.0002	299.8333	0.1248	692.1315	7.1578	81.7072
NGA	34.8400	134.2643	567.9307	68.0144	0.0027	299.8735	0.4332	610.3793	6.8835	76.9425
PRK	59.4120	190.4250	462.0000	23.1494	0.0029	279.5835	0.4881	438.4716	3.0490	244.1590
NOR	76.0200	12.2958	38,131.4606	6.6647	0.0038	276.1095	0.3001	455.3105	2.3991	69.1475
OMN	71.5690	7.3279	8601.2719	63.8336	0.0000	301.0708	0.0980	611.3024	5.8749	39.3910
PAK	32.9820	184.6508	576.1956	49.0836	0.0008	293.1199	0.1628	590.8792	2.2058	267.5996
PAN	62.1980	40.7632	4060.3178	13.6348	0.0089	297.2569	0.5840	394.5247	0.7364	244.9138
PNG	13.2040	12.9126	602.1865	9.0796	0.0106	296.5839	0.6776	327.0453	5.1675	112.5378
PRY	55.3310	13.3984	1663.6049	17.9527	0.0040	296.6231	0.6604	635.5005	2.0899	119.2261
PER	73.0420	20.6718	1955.5880	24.1718	0.0059	291.4839	0.5188	357.6134	2.4140	153.8799
PHL	46.1350	261.5681	1072.8018	18.4934	0.0086	298.6937	0.6469	412.0124	6.9327	75.0888
POL	61.7160	124.9098	4501.4541	22.6760	0.0020	282.7454	0.5454	562.3428	3.1817	233.2714
PRT	54.3990	112.4579	11,526.3721	10.2574	0.0028	288.0355	0.4980	543.3709	3.9791	269.3716
PRI	94.3870	429.6060	16,192.1270	7.6076	0.0023	298.2824	0.6881	670.5095	3.6219	72.3817
QAT	96.3110	51.0307	29,976.1676	80.0247	0.0002	300.3638	0.0733	469.5788	4.9598	39.7064
ROU	53.0040	97.7013	1659.9076	19.8515	0.0017	283.4783	0.5147	470.6392	4.5113	243.7243
RUS	73.3500	8.9490	1771.5941	8.7891	0.0015	267.7990	0.3076	416.1997	1.8080	223.8098
RWA	14.9260	321.5925	260.6077	34.7283	0.0039	291.8890	0.5219	571.9293	6.1396	89.7848
SAU	79.8480	9.6125	9171.3315	56.2450	0.0001	298.8600	0.0978	757.0784	5.4815	57.9364
SEN	40.3200	50.8894	613.7324	49.8031	0.0014	301.5726	0.3611	579.2657	6.4565	81.4271
SCG	55.6565	65.4590	1270.9292	22.7359	0.0016	284.7935	0.5190	522.1836	4.5595	241.4637
SLE	35.6260	63.5158	138.6987	26.1347	0.0070	298.6507	0.6086	463.2504	6.1708	75.8043
SGP	100.0000	6011.7716	23,852.3270	16.5333	0.0075	299.7349	0.3583	396.5966	0.0295	310.9979
SVK	56.2330	112.0316	5426.6243	22.8983	0.0023	282.2880	0.5555	488.4059	4.5387	238.1577
SVN	50.7540	98.7550	10,201.3035	19.5636	0.0036	283.2516	0.6602	407.7590	4.6899	240.2064
SLB	15.8130	14.7433	1017.3997	6.6717	0.0099	298.9251	0.7601	427.6718	7.3600	113.8153
SOM	33.2470	14.1426	138.0000	24.1472	0.0007	299.4842	0.2422	950.0417	5.2746	93.1439
ZAF	56.8910	37.0687	3374.7184	15.8417	0.0020	290.3620	0.3521	634.4547	1.4766	122.9264
KOR	79.6210	487.3327	12,256.9936	21.5060	0.0034	285.0842	0.5530	506.4678	3.5008	235.0233
ESP	76.2620	81.2983	14,749.6874	11.6369	0.0019	287.0505	0.4220	587.5106	4.1237	261.1680
LKA	18.3800	299.4356	869.6963	19.0157	0.0048	299.2747	0.6486	604.7394	1.5733	117.1275
SDN	32.4950	10.9920	366.1727	34.7812	0.0011	299.9408	0.2570	705.5234	7.3465	75.2464
SUR	66.4440	3.0189	2012.2817	12.6231	0.0052	298.5469	0.7257	517.6283	0.5135	112.1216
SWE	84.0260	21.6214	29,624.9127	6.2024	0.0026	277.2268	0.4447	532.5994	1.3699	89.5126
CHE	73.3830	181.7693	38,952.0342	14.2803	0.0047	279.5672	0.4802	348.6885	4.4889	244.4236
SYR	51.9470	89.2962	4910.7777	35.0439	0.0007	291.8141	0.1419	648.5038	1.2378	276.7992
TJK	26.5010	44.4150	138.4291	20.6490	0.0018	272.7145	0.1199	333.8860	0.9743	236.1803
TZA	22.3090	37.8180	410.9524	16.9708	0.0027	295.6497	0.4592	712.6197	6.0892	89.5988
THA	31.3860	123.2215	2007.7353	21.6337	0.0053	298.9750	0.5636	454.1186	5.9974	98.3362
BHS	82.0070	29.7747	27,098.1563	8.9429	0.0019	297.8790	0.4217	753.1452	1.1274	177.7540
GMB	47.8680	130.2083	594.1494	47.3966	0.0020	300.6097	0.4439	492.6517	6.5275	80.7165
TLS	24.2630	59.4732	415.0859	11.1325	0.0052	297.6300	0.6283	418.8668	0.5893	65.4982
TGO	32.9070	90.5388	302.9586	45.5305	0.0033	299.9210	0.5112	575.6099	6.5277	76.7960
TTO	23.0120	247.0096	6435.1342	16.1513	0.0037	298.9956	0.7225	656.3816	0.9006	95.0152
TUN	63.4320	62.4894	2211.8350	26.7292	0.0004	293.3065	0.1652	567.7196	2.4768	214.9042
TUR	64.7410	82.1696	4337.4780	27.5412	0.0018	284.2063	0.3164	508.6591	2.6696	262.8761
TKM	45.9130	9.6102	643.1754	38.3680	0.0004	289.8442	0.1026	592.8558	1.6951	261.2224
UGA	14.7860	118.3632	261.8691	26.2175	0.0037	296.1132	0.5103	571.3354	5.8946	100.5396
UKR	67.1450	84.8822	658.3486	18.6947	0.0018	282.4762	0.4637	554.5069	4.1270	244.4423
ARE	80.2360	44.1294	33,291.3663	63.9781	0.0000	300.9181	0.0994	538.2136	5.1435	33.8981
GBR	78.6510	243.4279	28,223.0676	10.5769	0.0034	282.5327	0.6028	660.2248	2.0750	228.4969
USA	79.0570	30.7973	36,329.9561	8.1234	0.0021	280.6254	0.3747	549.6992	1.0730	182.9310
URY	92.0280	18.9677	6875.0255	10.8090	0.0041	290.4875	0.6370	577.5936	0.6709	158.1804
UZB	46.1260	57.9464	558.2268	32.6027	0.0005	286.6472	0.1388	587.7854	0.9237	239.2938
VUT	21.6730	15.1734	1470.6359	7.8214	0.0083	298.2466	0.7490	565.9593	6.9639	109.6799
VEN	87.5590	27.4275	4842.0366	16.6986	0.0059	297.9843	0.6371	492.0027	0.6772	209.1847
VNM	24.3740	256.8971	390.0933	21.7131	0.0057	296.5575	0.5708	424.6761	5.5972	88.0971
YEM	26.2670	32.9736	554.4487	49.7422	0.0004	298.8508	0.1278	744.9897	7.6840	61.2422
ZMB	34.8020	14.0114	345.6896	19.4540	0.0033	294.8242	0.5256	722.4590	4.9860	87.8069
ZWE	33.7580	30.7134	563.0575	15.4038	0.0030	293.8599	0.4818	663.2904	3.9495	92.1134

**Table A2:** Year-wise mean value of all the parameters in 2010.

County	UR	PD	GDP_per	PM25	TP	T2M	NDVI	BLH	WS	WD
AFG	23.7370	44.7041	543.3065	40.7204	0.0009	286.4250	0.1258	582.9946	1.5712	238.5367
ALB	59.7830	18.7345	3497.9745	20.5700	0.0056	286.2711	0.4979	411.7556	4.2603	261.3849
DZA	52.1630	106.3146	4094.3484	37.7796	0.0002	297.8995	0.1255	782.0035	2.4855	135.5287
AGO	84.0870	120.3886	33,893.2773	27.3842	0.0033	295.5125	0.5584	606.2822	4.8289	86.5153
ARG	90.8490	14.9043	10,385.9644	16.5901	0.0019	287.1481	0.3549	704.5626	4.1713	196.0700
ARM	63.4400	101.0648	3218.3783	29.5612	0.0026	281.4223	0.3561	382.7114	3.7551	279.0243
AUS	85.1820	2.8679	52,087.9723	5.4536	0.0017	294.7844	0.3313	773.0299	1.6386	101.3626
AUT	57.3990	101.2874	46,903.7616	15.5127	0.0036	279.2503	0.4544	423.9552	3.7114	270.2746
AZE	53.4060	109.5423	5843.5338	29.3589	0.0013	288.2518	0.2954	409.4935	3.4296	293.1673
BGD	10.6420	337.8351	234.2355	65.4100	0.0063	298.9940	0.4803	452.3087	3.5927	85.0009
BLR	97.6510	359.8278	44,184.9464	18.9133	0.0022	280.0730	0.4540	504.5311	2.7671	265.3468
BEL	43.0930	81.5826	1036.5345	15.9062	0.0025	282.2197	0.5250	550.7787	2.0266	278.5898
BLZ	24.6330	57.0366	647.8361	16.4686	0.0039	298.7081	0.7285	567.6204	1.6053	230.9991
BEN	30.4620	1133.7131	781.1536	36.8136	0.0030	301.2797	0.5018	622.5555	7.4702	72.5202
BTN	72.3020	68.1245	6853.0029	21.1219	0.0099	280.5868	0.5184	270.8777	1.6595	89.9351
BOL	82.4270	35.4581	28,443.8885	34.8114	0.0035	293.7833	0.5590	606.6375	3.1015	162.1895
BIH	45.5580	72.3726	4635.5102	30.3548	0.0043	283.3393	0.5302	428.5856	4.4372	266.4097
BWA	74.6720	46.7414	6033.6862	23.4656	0.0015	295.2420	0.3574	725.7447	5.1183	99.7233
BRA	45.2280	14.1370	4304.1111	19.1522	0.0046	298.4231	0.6565	527.3657	1.5089	132.6561
BRN	66.4300	9.2759	1955.4602	8.5111	0.0086	300.1388	0.7268	350.6426	0.5654	87.0220
BGR	84.3350	23.4159	11,286.0715	23.9984	0.0024	284.7768	0.5340	443.5187	4.8532	260.0481
BFA	74.9610	73.7446	35,270.6421	37.8638	0.0019	302.3370	0.3541	665.5900	7.7266	73.5709
BDI	34.7930	17.9842	2258.1864	36.2426	0.0048	294.1609	0.5589	529.2802	6.5576	94.0927
KHM	62.4120	3.5063	6434.8125	19.4808	0.0047	301.0983	0.5857	528.4165	6.1715	69.9110
CMR	38.9040	7.0416	488.4217	39.3817	0.0045	298.2797	0.5566	514.8801	6.4620	84.5962
CAN	80.9370	3.7928	47,562.0834	4.0408	0.0016	269.5186	0.2325	418.8888	1.0153	226.3978
CAF	73.6070	198.0188	77,117.1260	30.2462	0.0037	299.2786	0.6538	569.4739	6.2528	84.2769
TCD	87.0740	22.9479	12,724.1041	47.5292	0.0009	301.2740	0.2104	711.4191	7.3593	74.7617
CHL	49.2260	141.9361	4550.4531	17.1131	0.0044	282.1471	0.3218	530.3233	6.4737	222.0735
CHN	47.3300	64.5690	1701.4757	33.5316	0.0022	279.8398	0.2917	562.5896	1.5989	224.9424
COL	51.5590	43.0311	1352.3023	19.6749	0.0100	297.4497	0.6436	334.4057	0.9222	123.3522
COM	40.0130	28.4792	334.0216	12.2778	0.0026	298.7503	0.6891	594.0341	6.9941	90.0638
COG	63.2560	12.5146	3076.5564	38.5241	0.0039	298.2270	0.6236	411.5523	5.6358	88.5784
COD	77.9640	40.7595	6336.7095	40.1152	0.0044	297.6502	0.7110	448.2185	5.8985	89.9481
CRI	27.9730	370.6051	1316.4912	17.5967	0.0130	296.7344	0.6700	357.9722	0.7398	225.9937
CIV	71.7360	89.6469	8227.1275	22.6921	0.0037	299.9743	0.5623	559.6354	6.9273	74.5711
HRV	76.5970	105.4663	5305.8836	20.5528	0.0039	284.4250	0.5424	440.8613	4.2722	267.6474
CUB	67.5510	120.4131	31,023.6387	9.7600	0.0028	298.1095	0.6683	664.9447	0.5620	194.1925
CYP	73.2550	135.6086	19,960.0685	22.8102	0.0010	293.8294	0.3730	511.1199	1.8997	273.7738
CZE	76.9660	234.6069	41,572.4560	20.1978	0.0026	280.3871	0.4437	504.3881	3.2755	275.2004
DNK	76.9990	36.2465	1343.2751	1.8124	0.0009	256.2492	−0.0304	209.7019	0.9164	148.4681
DJI	68.0940	94.5027	6967.3650	34.4946	0.0007	301.5621	0.1089	806.9927	7.5302	60.3222
DMA	86.7950	130.7491	58,041.3984	13.8750	0.0033	299.8107	0.7526	728.0241	1.3721	38.2577
DOM	73.7530	200.6855	5555.3920	12.5096	0.0035	297.4771	0.6906	484.9965	1.8477	112.2349
ECU	67.5400	15.1055	4480.7863	18.8808	0.0083	294.1817	0.5549	341.0703	0.5654	263.1343
EGY	62.6900	60.4409	4633.5913	48.1878	0.0000	297.6498	0.1154	601.6020	3.4849	79.6426
SLV	43.0190	83.1395	2645.9688	27.8099	0.0060	298.2016	0.6729	531.1998	1.1308	210.9113
GNQ	35.1750	31.3905	501.3553	34.2131	0.0072	297.5292	0.4614	374.2530	5.8789	89.9145
ERI	78.4420	93.1519	30,532.4805	34.7674	0.0009	300.5296	0.1592	710.7175	8.2645	66.4203
EST	68.0940	31.4101	14,663.0446	10.4435	0.0022	278.0834	0.4123	486.3156	0.8620	99.9942
ETH	17.3190	77.6088	341.5541	22.4263	0.0029	296.6997	0.3831	750.3512	5.8186	82.3852
FJI	83.7700	17.6484	46,505.3032	6.3517	0.0055	298.0352	0.7669	543.7714	6.7525	127.0238
FIN	52.1710	47.0616	3652.5359	6.6236	0.0018	274.2084	0.4011	485.6330	0.7865	92.5167
FRA	78.3690	118.7593	40,677.9851	14.5677	0.0026	283.5292	0.5657	550.2120	3.0208	266.7231
GAB	85.5330	6.3032	8849.3226	28.6259	0.0050	298.1025	0.4853	418.5210	5.4973	84.8032
GEO	81.3020	259.4402	39,688.6150	23.0475	0.0037	282.8617	0.4921	322.5517	4.1843	270.0103
DEU	55.5350	66.2496	3233.2959	15.0321	0.0026	281.2116	0.4723	552.1991	2.5113	287.3649
GHA	50.7130	108.9022	1299.3452	28.2610	0.0032	301.0210	0.5140	574.2501	7.0283	73.9627
GRC	33.6780	41.4788	672.4249	17.8012	0.0027	288.3139	0.5350	487.4592	3.8180	257.7429
GTM	55.6620	177.1936	860.6364	23.6332	0.0060	296.2301	0.6984	460.7317	1.6965	211.6805
GIN	40.1110	54.1466	558.1747	29.9936	0.0047	299.5000	0.5690	554.3768	7.3328	73.9678
GNB	65.9400	33.6414	17,288.8413	34.3831	0.0034	301.2685	0.5760	506.7190	7.2528	75.1912
GUY	76.2920	86.2788	26,716.6488	19.2081	0.0063	298.5927	0.6857	436.3221	1.3986	83.1539
HTI	48.4030	133.0691	2852.5473	13.6283	0.0035	298.5115	0.6289	482.3432	2.5021	108.7013
HND	26.6340	3.8071	4580.6988	23.7494	0.0054	296.6075	0.7097	459.8782	1.3912	242.3355
HUN	51.8850	74.3361	1904.3472	19.8155	0.0029	283.6719	0.4845	461.2708	4.2378	269.3359
ISL	55.1550	76.7589	14,067.5231	5.1105	0.0029	275.2911	0.1557	474.0290	0.4757	176.7534
IND	47.5090	361.0057	1191.9727	45.5661	0.0039	297.2297	0.4219	558.1978	1.6909	171.6800
IDN	68.9110	110.4609	13,223.0830	13.8318	0.0104	298.5568	0.7013	303.7251	1.4201	96.7373
IRN	49.9140	133.4943	3122.3627	41.0969	0.0006	292.3816	0.1351	714.8023	4.3480	308.9607
IRQ	30.9300	415.1370	1357.5637	56.5689	0.0004	297.6370	0.1351	697.2399	2.0903	268.1486
IRL	61.5420	66.1947	48,655.3662	9.2998	0.0025	281.7896	0.6377	605.3116	0.7395	278.0849
ISR	70.6260	45.2875	6599.6609	27.3870	0.0006	294.8444	0.2070	490.9759	0.8147	126.4837
ITA	69.1030	68.4794	4657.2803	16.5809	0.0037	285.2070	0.5284	421.8136	3.7691	264.5229
JAM	93.5740	3.1725	43,237.0730	16.8277	0.0042	298.7094	0.7376	518.1663	0.5266	123.4435
JPN	91.8260	352.2921	30,780.0238	10.3896	0.0052	285.1543	0.5632	537.7658	2.9139	242.7836
JOR	68.3270	201.5279	36,035.6450	33.6382	0.0002	294.4903	0.1226	655.6845	0.9674	164.3673
KAZ	53.7430	259.5073	4704.0478	17.1464	0.0008	280.3791	0.1589	574.3142	1.0340	234.9184
KEN	86.0880	81.7925	3736.6455	18.7693	0.0020	298.6075	0.3634	887.2977	6.0978	108.3308
KWT	90.8120	351.3580	44,968.1562	55.6635	0.0001	300.5263	0.0848	676.3921	2.7809	16.7101
KGZ	56.8270	6.0458	9070.4883	23.1768	0.0029	274.8617	0.1842	273.0803	1.6108	242.2357
LAO	23.5710	73.8495	1080.2962	31.7077	0.0052	296.6715	0.6831	428.5346	4.3811	73.6964
LVA	35.3060	28.4041	880.0378	16.8554	0.0024	278.7494	0.4569	504.1107	1.3196	292.6289
LBN	20.2940	81.0798	785.5027	29.4808	0.0013	292.1864	0.3321	454.8625	1.4076	292.7077
LSO	81.9360	509.8160	23,087.2256	30.9382	0.0029	285.4497	0.3767	618.2869	2.3187	101.5551
LBR	100.0000	167.8947	38,577.4983	20.8104	0.0069	298.3473	0.5726	405.1455	6.4145	76.4804
LBY	30.0640	27.0761	1141.2357	44.9146	0.0000	297.0999	0.1089	639.5184	2.9280	99.6819
LTU	87.3340	484.1705	7761.6415	18.8983	0.0024	279.3566	0.4402	519.3005	2.1780	299.6535
LUX	47.8130	40.4003	513.4456	14.5062	0.0026	280.9109	0.5379	537.4608	2.4119	274.8031
MKD	78.0520	3.5223	12,162.6687	28.5769	0.0027	284.4014	0.5094	489.8313	4.4800	261.3908
MDG	18.2260	323.1022	2799.6487	14.2395	0.0037	296.2955	0.4989	612.5914	5.9437	87.6547
MWI	24.7980	65.7304	1119.8436	23.1781	0.0035	295.1841	0.4354	583.2161	5.9511	86.2580
MYS	66.7570	49.4181	11,987.5084	11.8684	0.0085	299.0985	0.7260	322.4270	1.0603	164.5732
MLI	88.5470	208.6226	110,885.9914	43.6481	0.0008	302.9405	0.1930	734.4775	6.4881	78.4974
MLT	67.8410	33.7011	11,420.9940	14.7000	0.0010	292.4508	0.2650	644.2999	2.3978	252.1285
MRT	58.0180	72.4701	2839.9260	49.4271	0.0002	301.7911	0.1163	667.7209	5.5606	80.9139
MUS	42.6200	99.7486	2437.5377	14.9643	0.0018	297.6239	0.6674	832.2022	6.3819	91.2177
MEX	31.9380	36.3718	471.9592	10.7505	0.0025	293.5143	0.4538	622.6082	1.0130	242.3290
MDA	77.8150	58.6913	9271.3984	17.1430	0.0021	283.4861	0.4243	511.0675	4.9533	261.5537
MNG	57.0890	81.4831	4577.6888	20.3286	0.0007	273.3294	0.1509	605.1658	1.1409	292.6526
MAR	35.9990	12.3336	710.2743	23.8466	0.0013	291.8975	0.2227	635.2521	1.2974	212.5027
MOZ	94.0720	1295.3375	21,799.1743	21.1780	0.0024	297.3460	0.5703	681.6963	5.5648	90.3917
MMR	28.8850	77.4589	746.9454	32.3807	0.0063	297.0950	0.5897	425.0941	3.4974	70.7156
NAM	67.5670	1.7508	2643.2871	17.8317	0.0011	294.6526	0.2440	706.3795	4.9635	100.6480
NPL	31.8300	29.9239	471.9044	33.6434	0.0058	285.5679	0.4564	352.1509	2.0649	125.1047
NLD	46.5880	3.3901	1610.9206	15.4084	0.0024	282.1098	0.5103	561.5207	1.7501	294.1775
NZL	41.5550	615.9606	8000.3764	7.6132	0.0050	283.7062	0.6549	537.2214	5.1672	278.0932
NIC	15.5440	154.2173	478.6687	17.8166	0.0056	298.5502	0.6385	554.3769	1.0923	236.3933
NER	70.9120	85.8561	9040.5685	61.9910	0.0003	301.7717	0.1266	706.7643	6.9274	78.2103
NGA	41.6160	2.5737	5394.9967	54.4786	0.0030	300.9623	0.4178	611.8713	7.4695	74.0082
PRK	16.2210	12.9976	476.8695	22.1498	0.0039	279.6718	0.4537	457.9945	3.6046	257.7851
NOR	43.4800	174.0321	2280.4373	6.2609	0.0030	273.7207	0.2744	397.2826	2.4269	39.2756
OMN	56.9170	48.3967	1503.8722	54.1434	0.0001	301.6586	0.1020	634.2790	5.6934	35.3926
PAK	87.1340	492.5999	50,999.7451	50.6556	0.0013	293.5166	0.1867	552.6995	2.3629	265.0578
PAN	79.1020	13.3863	87,693.7901	14.9683	0.0112	298.2721	0.6080	377.9468	0.7914	191.1319
PNG	16.7680	188.4423	592.4012	14.3755	0.0110	297.0078	0.7099	320.3813	5.4797	117.6067
PRY	86.1600	16.5231	33,676.7741	17.6271	0.0034	296.7699	0.6201	662.0198	2.1740	124.1355
PER	75.1610	9.8269	21,369.3530	29.1773	0.0053	292.2713	0.5492	380.9177	2.8653	173.1772
PHL	34.9970	232.7530	987.4097	19.1501	0.0070	299.4058	0.6799	428.2899	5.6060	81.2701
POL	65.1400	49.0004	8082.0162	25.3577	0.0026	280.6870	0.4420	514.8395	3.0274	283.8089
PRT	76.4300	22.6779	5082.3537	8.3592	0.0030	288.2484	0.5331	555.1662	2.6697	268.5799
PRI	45.3320	315.1450	2217.4722	6.3975	0.0040	299.0158	0.7279	582.3037	3.2937	91.3208
QAT	13.0190	16.1430	1949.3512	77.0309	0.0001	301.2879	0.0789	432.1830	4.5870	35.5800
ROU	60.8920	124.2092	12,613.0110	18.2238	0.0027	282.9693	0.5020	410.9185	4.9553	263.3766
RUS	93.8250	419.5631	26,435.7488	8.9189	0.0015	267.9939	0.2840	439.2652	1.5646	181.0101
RWA	60.3770	203.8771	571.0000	37.3556	0.0047	292.6306	0.5793	527.0604	6.2825	94.5689
SAU	59.2610	15.7262	4342.0658	62.9081	0.0001	300.0993	0.0997	788.9404	4.8292	59.2396
SEN	98.5010	159.8905	67,403.0877	42.2514	0.0015	302.3778	0.3715	584.6552	7.0620	77.3037
SCG	53.8290	88.0107	8214.0769	23.0210	0.0031	284.1093	0.5089	461.0485	4.6190	264.2004
SLE	73.6870	8.7226	10,674.9961	25.4695	0.0068	299.0767	0.6069	454.3014	6.9283	73.9357
SGP	16.9340	406.9452	609.7543	12.7667	0.0077	300.3449	0.3782	382.4692	0.0733	266.9524
SVK	82.0840	12.7560	19,262.5476	22.4561	0.0032	281.0620	0.5077	448.5882	3.8797	272.7194
SVN	59.5665	64.7115	6211.9526	18.5665	0.0041	282.1086	0.5702	409.4799	4.0335	268.6721
SLB	33.0890	13.9218	1683.2119	9.4798	0.0101	299.4975	0.7754	473.4059	7.5332	116.0296
SOM	43.7730	65.8502	1271.5833	20.5293	0.0006	300.1017	0.2314	962.1868	5.2201	95.2566
ZAF	100.0000	7231.8120	47,236.9602	20.4826	0.0015	291.3567	0.3396	687.0730	2.5442	97.3376
KOR	20.0480	18.8589	1604.1489	21.4609	0.0033	285.1610	0.5397	509.4174	4.2969	251.6809
ESP	38.8560	88.8838	401.8349	9.9437	0.0023	286.5493	0.4453	592.2915	2.7447	263.7488
LKA	65.4520	298.4497	2983.2288	25.3155	0.0060	299.8506	0.6618	586.3166	1.3345	166.5525
SDN	39.3100	19.1983	340.0000	38.9988	0.0011	301.3470	0.2519	738.0433	7.3270	74.6513
SUR	64.9520	187.8875	1090.2608	16.9544	0.0063	299.2193	0.7294	472.5307	2.1512	82.3240
SWE	66.3440	3.3918	8255.8749	5.7732	0.0021	274.3062	0.3890	469.8716	1.5591	71.7317
CHE	54.6850	112.1089	16,841.7677	14.9906	0.0045	278.4398	0.3945	363.3662	3.4651	266.6112
SYR	52.6580	101.6858	23,532.4809	40.9309	0.0005	293.6260	0.1708	645.8788	2.0639	292.5738
TJK	85.0560	22.8545	52,869.0443	21.2128	0.0022	273.5668	0.1295	293.0872	0.9914	233.3864
TZA	55.6000	116.3347	1182.6078	23.7108	0.0027	296.2039	0.4929	700.7487	6.6704	92.9072
THA	21.9850	9.4918	892.5689	27.1362	0.0046	299.9986	0.5820	490.1417	4.6816	99.8046
BHS	37.5330	118.0672	534.0448	7.7060	0.0028	297.7795	0.4434	807.5737	1.2382	185.8730
GMB	43.8560	131.5254	5076.3399	40.4773	0.0023	301.3224	0.4435	489.6556	7.1401	76.6141
TLS	26.5200	53.7825	749.5524	11.6718	0.0056	298.3395	0.6927	383.0494	0.8008	89.9006
TGO	48.4910	10.8255	4439.2021	32.3445	0.0035	300.6441	0.5188	549.1817	7.1811	72.9087
TTO	27.7320	73.5385	806.4143	18.2077	0.0043	300.0594	0.7054	621.9412	2.2701	65.9014
TUN	54.0250	258.8975	16,683.3931	26.6958	0.0006	293.7851	0.1735	608.7419	1.5471	233.4501
TUR	66.6570	68.4555	4344.6194	30.0736	0.0022	286.3770	0.3624	541.6698	3.8240	268.8586
TKM	70.8250	93.9763	10,742.7750	35.6079	0.0004	290.5273	0.1137	568.9996	1.6084	258.3359
TCA	28.1140	50.0638	743.4037	6.5000	0.0047	296.4078	0.2566	522.6992	6.0258	105.3096
UGA	19.3830	161.7203	822.5394	28.7182	0.0019	282.6911	0.5464	534.6531	4.3630	258.9520
ARE	68.5960	79.1803	3078.4299	60.7467	0.0001	301.9237	0.1055	529.0540	4.9619	29.6262
GBR	94.4140	19.1937	11,992.0238	10.6468	0.0024	281.3542	0.5298	587.7255	1.1419	220.0884
USA	80.7720	33.8158	48,650.6431	6.5162	0.0021	280.7282	0.3657	546.2695	1.1354	181.6406
URY	50.9560	67.1425	1742.3493	10.1519	0.0035	290.6031	0.6071	551.7682	1.3857	120.9040
UZB	88.0830	32.2430	13,825.3571	32.7840	0.0006	287.1962	0.1537	568.6717	0.9337	230.7533
VUT	30.4170	283.7026	1673.3293	8.6629	0.0057	298.5864	0.7746	605.4128	6.8839	109.8597
VEN	24.4620	19.3779	2839.4063	20.2724	0.0073	299.1591	0.6504	449.7746	1.4295	85.8965
VNM	31.7760	43.8564	1334.7849	28.2711	0.0059	297.3987	0.5967	448.5403	4.5158	89.2613
YEM	62.2180	42.2203	8148.9612	44.7159	0.0005	299.2449	0.1327	764.0328	7.6672	57.8002
ZMB	39.3550	18.3026	1489.4591	26.0485	0.0033	295.2124	0.5530	695.4833	5.4526	87.0685
ZWE	33.1960	32.8234	948.3315	20.7320	0.0022	295.0207	0.4670	698.6781	5.3556	94.2311

**Table A3:** Year-wise mean value of all the parameters in 2019.

Country	UR	PD	GDP_per	PM25	T2M	TP	NDVI	BLH	WS	WD
AFG	25.7540	58.2694	494.1793	38.0847	285.9910	0.0012	0.1220	576.7270	1.1174	192.4120
ALB	61.2290	104.1676	5396.2159	18.4450	287.0500	0.0037	0.5194	420.1060	0.5200	86.7770
DZA	73.1890	18.0763	3989.6683	30.8674	296.9940	0.0002	0.1241	804.5470	1.3350	112.8540
AGO	66.1770	25.5276	2177.7990	22.8370	296.4210	0.0029	0.5436	643.1190	0.9093	149.4730
ARG	91.9910	16.4208	10,076.3552	15.2006	287.6710	0.0019	0.3775	695.4480	1.7135	183.4740
ARM	63.2190	103.8893	4604.6463	25.2962	280.2240	0.0021	0.3240	401.6270	0.3271	152.5670
AUS	86.1240	3.2977	54,875.2860	6.7456	296.1620	0.0007	0.2789	888.7900	1.7871	140.7900
AUT	58.5150	107.6093	50,114.4011	10.2758	281.2490	0.0035	0.5170	441.2720	0.5876	262.1070
AZE	56.0310	121.2801	4805.7537	24.6990	287.5710	0.0012	0.2915	445.8150	1.1048	114.1380
BGD	37.4050	1252.5634	2154.2268	66.1645	298.7430	0.0057	0.5475	429.5680	0.6691	142.3130
BLR	79.0440	46.4073	6837.7178	12.7372	281.9500	0.0018	0.5131	597.0160	0.9863	236.0810
BEL	98.0410	379.4247	46,599.1113	9.6846	284.1720	0.0025	0.6249	595.0370	1.5952	222.0780
BLZ	45.8660	17.1132	4983.3361	16.5686	299.5650	0.0028	0.7292	630.8900	1.9661	82.6295
BEN	47.8610	104.6572	1219.5155	38.4915	301.1550	0.0033	0.4941	601.6600	0.8456	188.9890
BTN	41.6120	20.0077	3322.8633	22.2679	280.0860	0.0085	0.5505	268.1330	0.3853	180.7190
BOL	69.7730	10.6278	3552.0681	28.5346	293.8780	0.0041	0.5769	558.7200	0.7500	223.5320
BIH	48.6260	64.4726	6119.7624	24.1778	284.5840	0.0035	0.5902	433.3170	0.3310	146.7670
BWA	70.1720	4.0649	7247.4295	17.6292	296.5670	0.0009	0.2981	841.4250	1.9023	67.4814
BRA	86.8240	25.2508	8876.0598	17.7093	298.6320	0.0046	0.6579	527.5280	1.1408	82.4332
BRN	77.9420	82.2194	31,085.9619	11.1185	300.5500	0.0064	0.7576	366.5130	0.2535	184.3600
BGR	75.3470	64.2572	9879.2685	20.2587	285.7290	0.0018	0.5366	473.0070	0.4082	163.8010
BFA	29.9800	74.2741	796.1152	33.3853	302.1930	0.0017	0.3440	651.9520	0.3447	165.9440
BDI	13.3660	449.0100	223.8629	33.3833	294.2130	0.0054	0.5516	491.9110	0.7624	155.1150
KHM	23.8050	93.3976	1643.1214	19.0451	301.2210	0.0043	0.5770	579.4180	0.6272	203.3360
CMR	56.9680	54.7405	1533.0957	39.5674	298.1940	0.0053	0.5745	496.3120	0.7571	231.8490
CAN	81.4820	4.1939	46,328.6718	3.7887	267.7350	0.0017	0.2210	415.1060	1.0458	232.4510
CAF	41.7700	7.6169	468.1175	29.9804	299.7250	0.0032	0.6687	607.9740	0.5128	190.3200
TCD	23.2790	12.6643	709.5400	45.7528	300.7440	0.0009	0.2158	694.0220	1.8188	99.1951
CHL	87.6430	25.4892	14,699.4628	15.6574	282.9200	0.0042	0.3270	513.9030	1.6353	261.0210
CHN	60.3080	149.3676	10,143.8382	25.5362	280.2820	0.0020	0.3143	548.3210	1.0782	193.2240
COL	81.1040	45.3713	6418.6158	20.0078	297.5740	0.0085	0.6356	355.1230	0.5836	125.3620
COM	29.1640	457.2225	1404.4332	11.4444	298.9590	0.0037	0.6562	563.6050	1.2153	152.7340
COG	67.3730	15.7555	2369.7294	35.3891	298.4350	0.0045	0.6474	402.2990	0.9475	238.3690
COD	45.0460	38.2835	596.5606	37.0104	297.9160	0.0043	0.7050	450.7590	0.5152	189.8170
CRI	80.0760	98.8555	12,762.1380	16.5802	296.9290	0.0077	0.6854	402.6680	1.6485	95.5486
CIV	51.2390	80.8697	2276.3324	22.0734	299.8870	0.0039	0.5649	557.8230	1.1970	205.1350
HRV	57.2420	71.8369	15,311.7669	15.6601	286.0370	0.0034	0.5945	442.2140	0.5484	139.2350
CUB	77.1090	109.1858	9125.8787	10.1175	299.2330	0.0026	0.6975	619.1490	2.1725	73.5690
CYP	66.8050	129.7158	29,206.0762	17.3184	293.4220	0.0020	0.4344	514.9480	1.1033	280.8170
CZE	73.9210	138.2367	23,660.1488	12.3749	282.9200	0.0021	0.5658	558.9770	0.8069	252.8090
DNK	87.9940	145.3606	59,775.7351	1.8211	255.3890	0.0008	−0.0289	199.5540	4.5992	196.7030
DJI	77.9150	41.9999	3172.7507	36.1669	302.1320	0.0005	0.1061	833.7160	1.7137	109.2990
DMA	70.7860	95.7440	8516.2800	10.8750	299.3400	0.0023	0.7133	760.6850	7.0991	82.9319
DOM	81.8280	222.2926	8282.1171	12.3714	297.8290	0.0023	0.6724	540.1800	1.7639	99.3554
ECU	63.9860	69.9535	6222.5247	16.5185	294.3210	0.0097	0.5203	327.1850	0.8564	177.1230
EGY	42.7300	100.8469	3019.0923	45.6146	296.3580	0.0000	0.1163	647.5970	2.2996	237.8120
SLV	72.7460	311.4648	4167.7309	28.4136	299.2960	0.0039	0.6899	609.6880	0.6856	99.3884
GNQ	72.6270	48.3416	8380.7411	32.5806	297.8800	0.0091	0.5520	368.3300	1.1905	245.0380
ERI	88.7850	34.6249	879.7500	35.2604	300.4940	0.0011	0.1773	683.8020	0.7730	127.0570
EST	69.0510	30.5235	23,397.8783	6.6522	280.5120	0.0022	0.4676	577.9460	1.4355	232.5920
ETH	21.2250	99.2462	855.7609	21.4045	297.0090	0.0031	0.3999	738.1400	0.9869	146.1410
FJI	56.7500	48.7113	6175.8907	6.4917	298.0460	0.0075	0.7729	528.3190	2.4432	117.4620
FIN	85.4460	18.1680	48,628.6418	4.1757	275.9150	0.0021	0.3976	514.1460	0.5794	248.4900
FRA	80.7090	122.8163	40,578.6443	8.6285	285.2670	0.0027	0.6039	574.8230	0.9587	244.9160
GAB	89.7410	8.4316	7766.9966	27.3496	298.3740	0.0063	0.5362	401.3820	1.0735	237.0970
GEO	59.0390	65.0856	4696.1506	20.5521	281.8770	0.0030	0.4900	329.1450	0.3831	166.2410
DEU	77.3760	237.8298	46,794.8993	9.6144	283.6320	0.0023	0.6098	589.4600	1.2873	238.8680
GHA	56.7070	133.6814	2246.6256	28.3200	301.1960	0.0036	0.5091	568.7270	1.2022	203.5150
GRC	79.3880	83.1775	19,133.7578	15.4170	288.4520	0.0025	0.5523	504.4960	0.6131	177.9470
GTM	51.4390	154.9461	4638.6349	24.2976	296.9800	0.0042	0.7107	520.3380	0.8342	94.0165
GIN	36.5000	51.9748	1052.5881	26.8087	299.7110	0.0047	0.5822	566.5580	0.6415	228.1720
GNB	43.7770	68.3114	749.4537	30.4515	301.1960	0.0031	0.5734	518.2760	0.8025	271.5200
GUY	26.6890	3.9765	6609.5113	16.6437	298.7880	0.0056	0.6699	503.5820	1.7712	51.5988
HTI	56.1920	408.6749	1312.7706	13.0648	298.9520	0.0026	0.6046	486.0390	1.3718	71.3106
HND	57.7300	87.1044	2574.3568	25.1231	297.5060	0.0037	0.6992	543.1380	1.2021	63.4658
HUN	71.6440	107.0693	16,735.6598	14.7373	285.6490	0.0020	0.5244	508.8610	0.3774	281.7820
ISL	93.8550	3.5759	68,941.4622	4.6877	275.1510	0.0035	0.1591	499.5160	1.3305	95.8075
IND	34.4720	459.5797	2072.2449	47.7395	297.0040	0.0039	0.4375	570.4330	0.6883	227.2730
IDN	55.9850	144.1400	4135.2333	19.3366	298.7120	0.0080	0.6920	366.1870	0.4894	157.6720
IRN	75.3910	50.9061	3514.0422	39.2695	291.6150	0.0011	0.1417	706.7170	0.9124	184.8040
IRQ	70.6780	90.5488	5943.4585	41.0859	296.4490	0.0009	0.1950	653.7620	1.4538	277.4360
IRL	63.4050	71.6264	80,886.6157	8.3900	283.1850	0.0033	0.7052	683.9150	1.5995	229.9180
ISR	92.5010	418.3919	43,951.2477	24.7428	293.7420	0.0009	0.2257	531.0790	1.2911	294.0130
ITA	70.7360	200.6149	33,673.4755	12.8533	286.4040	0.0033	0.5514	422.3290	0.5586	193.7010
JAM	55.9850	272.2324	5369.4984	16.7798	299.6320	0.0031	0.7483	556.2090	2.2790	86.7641
JPN	91.6980	347.4156	40,458.0019	10.0334	285.4470	0.0046	0.5885	536.0390	0.8988	263.3480
JOR	91.2030	113.7835	4405.4871	29.7442	293.1010	0.0003	0.1269	691.4660	1.5888	284.3500
KAZ	57.5400	6.8577	9812.5958	15.8898	280.8730	0.0008	0.1711	578.5250	0.9060	150.6460
KEN	27.5070	92.3744	1909.3045	17.3898	299.0080	0.0024	0.3672	880.3930	2.5551	128.7880
KWT	100.0000	236.0874	32,373.2511	49.3006	299.8690	0.0004	0.0944	705.1380	2.0729	315.0530
KGZ	36.5910	33.6611	1374.0321	20.9686	275.0740	0.0024	0.1847	318.9340	0.6110	174.5800
LAO	35.6450	31.0635	2613.9444	28.1527	297.2150	0.0043	0.6666	478.0500	0.5116	155.9140
LVA	68.2220	30.8234	17,926.8416	11.4510	281.2190	0.0021	0.5086	601.8390	1.3511	229.9350
LBN	88.7580	670.1573	7527.4431	28.1058	291.2930	0.0023	0.3510	472.8410	1.2080	228.9240
LSO	28.5850	70.0022	1153.3881	25.2854	286.5400	0.0021	0.3611	713.2940	1.1283	296.3580
LBR	51.6150	51.2601	672.3405	20.7036	298.8470	0.0076	0.5940	426.6500	1.1926	228.4800
LBY	80.3930	3.8518	10,218.0430	38.7454	295.7350	0.0001	0.1101	676.7930	1.6116	113.0800
LTU	67.8550	44.6134	19,575.7685	12.6261	281.9140	0.0018	0.4940	625.8760	1.1782	227.3760
LUX	91.2230	255.1444	113,218.7133	8.3344	282.8150	0.0028	0.6410	563.4620	1.1463	227.9150
MKD	58.2080	82.3431	6070.3881	24.0747	285.1600	0.0019	0.4955	531.3080	0.4045	238.2810
MDG	37.8610	46.3549	526.2246	12.1826	296.4980	0.0040	0.5163	583.1610	1.2896	127.2640
MWI	17.1740	197.5896	591.8471	21.9481	295.3550	0.0043	0.4373	573.6070	1.7421	124.7420
MYS	76.6070	97.2448	11,432.8260	16.0935	299.4560	0.0068	0.7413	355.8280	0.2674	119.3260
MLI	43.1360	16.1106	879.0432	37.2800	301.9070	0.0007	0.1943	737.5190	1.2539	59.9142
MLT	94.6780	1575.1938	31,185.6496	12.5500	292.6660	0.0015	0.2972	636.0850	2.0472	296.7260
MRT	54.5070	4.3909	1743.3013	47.0931	300.3140	0.0002	0.1110	675.0430	2.4489	53.1952
MUS	40.7660	623.5030	11,097.1690	14.9857	297.9390	0.0022	0.6978	798.3020	4.4339	107.4560
MEX	80.4440	65.6270	9950.2176	11.4090	294.4040	0.0020	0.4537	641.2160	1.0560	176.7620
MDA	42.7260	92.7841	4492.1057	14.4353	284.9030	0.0013	0.4343	560.0100	0.3871	302.3710
MNG	68.5430	2.0711	4404.8458	16.9930	274.8340	0.0007	0.1783	592.7250	1.2935	270.1860
MAR	62.9940	81.7203	3235.0007	19.8356	291.6200	0.0006	0.2021	628.2860	1.1931	248.2390
MOZ	36.5280	38.6150	506.8171	19.1836	297.5160	0.0028	0.5734	677.4150	1.4815	129.0600
MMR	30.8520	82.7914	1271.1115	31.3504	297.0020	0.0056	0.6225	428.0970	0.5503	203.3390
NAM	51.0420	3.0299	5028.2953	13.7310	296.2790	0.0005	0.1939	792.7700	1.2406	135.3750
NPL	20.1530	199.5725	1194.9572	35.3453	284.8090	0.0060	0.4904	317.6700	0.2982	199.8580
NLD	91.8760	515.1433	52,476.2733	10.3351	284.2960	0.0024	0.6263	614.1070	1.6971	229.9490
NZL	86.6150	18.9100	42,865.2336	8.2814	284.0760	0.0049	0.6630	564.6350	1.4978	291.8250
NIC	58.7600	54.3917	1924.4718	17.4163	299.0100	0.0043	0.6269	614.1830	2.3357	56.4603
NER	16.5170	18.4027	554.0994	52.5541	301.0240	0.0003	0.1285	716.1880	1.5739	73.4185
NGA	51.1570	220.6524	2229.8587	56.4806	300.4600	0.0033	0.4262	572.1060	0.8015	194.9070
PRK	62.1340	213.1564	31,902.2435	19.0533	281.0850	0.0023	0.5019	484.3070	0.9032	278.8070
NOR	82.6160	14.6474	75,719.7529	5.2319	275.7580	0.0037	0.2848	428.8400	0.6042	215.2790
OMN	85.4430	16.0743	17,700.7035	57.6261	301.5860	0.0001	0.1000	662.1160	1.2113	169.2910
PAK	36.9070	280.9326	1481.8139	48.5352	292.9330	0.0014	0.2009	571.4580	0.8369	193.6570
PAN	68.0590	57.2474	15,774.2550	14.8707	298.4130	0.0073	0.6259	389.2420	1.3280	189.5370
PNG	13.2500	19.3793	2820.3064	12.4012	296.7420	0.0118	0.6730	345.0370	0.5988	187.4480
PRY	61.8790	17.7313	5383.5744	17.8334	297.7010	0.0035	0.6478	645.6430	0.9680	79.5810
PER	78.0990	25.3988	7023.0775	26.7666	292.1290	0.0065	0.5226	361.7310	0.6057	164.2370
PHL	47.1490	362.6006	3485.3408	19.4999	299.4700	0.0064	0.7016	476.7210	0.6597	106.2000
POL	60.0370	124.0013	15,732.2031	16.3124	283.4590	0.0018	0.5542	603.7460	1.1526	242.8570
PRT	65.7640	112.2886	23,330.8173	6.9603	288.3190	0.0021	0.5310	552.3540	1.4215	305.3360
PRI	93.5760	360.0557	32,850.5486	6.5975	299.0080	0.0024	0.7144	608.7930	2.9053	88.8691
QAT	99.1880	246.4814	62,087.9741	84.4423	301.1310	0.0003	0.0796	487.5470	1.9445	349.3780
ROU	54.0840	84.1953	12,899.3461	15.8196	284.3510	0.0020	0.5149	440.4420	0.5124	169.2870
RUS	74.5870	8.8177	11,536.2510	9.5408	269.5330	0.0016	0.2893	446.3880	1.0627	212.2090
RWA	17.3130	511.8337	820.1772	35.1657	292.7650	0.0049	0.5440	494.8830	0.5002	147.2600
STP	73.5980	224.0083	1987.5797	15.5200	299.1360	0.0040	0.5494	482.4470	3.9310	201.5850
SAU	84.0650	15.9411	23,450.5620	61.9292	299.7860	0.0002	0.1054	804.9950	1.0991	169.7930
SEN	47.6530	84.6432	1435.8304	41.3382	302.0240	0.0011	0.3343	608.2480	1.3218	323.5690
SLE	42.4840	108.2461	521.7548	25.2171	299.5970	0.0081	0.6111	477.3480	1.1576	227.5600
SGP	100.0000	8044.5261	65,831.1894	18.9333	300.6100	0.0064	0.4149	441.5890	0.7305	77.1261
SVK	53.7290	113.4390	19,303.5457	15.3297	282.9830	0.0024	0.5620	496.2970	0.3968	191.0500
SVN	54.8220	103.7119	25,942.9548	12.9523	283.9230	0.0040	0.6477	394.5650	0.3443	196.2730
SLB	24.2100	23.9307	2344.0490	10.1254	299.5710	0.0117	0.7169	475.2730	1.2920	133.6140
SOM	45.5540	24.6165	419.3948	22.1128	300.1780	0.0011	0.2472	898.5060	2.0717	170.4720
ZAF	66.8560	48.2720	6624.7619	16.9129	292.2730	0.0011	0.3138	722.0870	0.9336	202.9530
KOR	81.4300	530.8124	31,902.4169	20.0633	286.0840	0.0031	0.5821	488.9350	0.8403	290.4310
ESP	80.5650	94.3445	29,554.4905	8.5732	287.5050	0.0018	0.4497	623.3620	0.8944	269.4560
LKA	18.5850	352.4344	3848.2124	22.6617	300.0680	0.0046	0.6573	607.7990	1.1082	224.2200
SDN	34.9360	23.1519	755.3290	35.9188	300.8210	0.0012	0.2713	730.9470	1.6993	112.8630
SUR	66.0950	3.7267	6853.6934	15.5669	299.4120	0.0052	0.7282	561.0120	2.0139	53.1437
SWE	87.7080	25.2360	51,939.4297	4.7841	276.6110	0.0024	0.4247	517.3740	0.7624	262.2180
CHE	73.8490	217.0076	85,334.5195	9.4789	280.1400	0.0046	0.4526	353.3350	0.3907	212.1300
SYR	54.8210	92.9594	1116.0000	33.6416	292.6530	0.0010	0.2275	625.9540	1.6093	264.8740
TJK	27.3090	67.1592	890.5444	20.0940	273.9880	0.0019	0.1264	366.9720	0.6048	166.8300
TZA	34.5000	65.4837	1085.8849	21.9575	296.3970	0.0034	0.5043	654.8030	1.4502	115.6950
THA	50.6920	136.2829	7814.3844	25.3366	300.1650	0.0039	0.5937	536.7530	0.5997	191.6000
BHS	83.1320	38.9097	33,872.3343	8.0357	298.9800	0.0031	0.4323	711.3870	3.0489	97.5827
GMB	61.9310	231.9858	772.5056	39.1057	300.8000	0.0017	0.4342	489.6130	1.3197	312.5200
TLS	30.9470	86.9617	1583.7136	11.8034	298.0910	0.0036	0.6329	470.7030	0.7706	133.0200
TGO	42.2480	148.6001	893.3525	33.1021	300.7360	0.0039	0.5133	539.2930	0.9114	194.8140
TTO	53.1870	271.9238	17,123.1163	14.6974	299.7350	0.0030	0.7140	695.1390	4.4938	84.0839
TUN	69.2540	75.2750	3571.9450	23.0214	293.4340	0.0007	0.1920	627.1390	1.0385	176.1760
TUR	75.6300	108.4022	9121.5152	28.3322	285.6730	0.0020	0.3548	543.2650	0.5421	159.6310
TKM	52.0480	12.6446	7612.0352	29.2526	290.4090	0.0006	0.1424	575.1930	1.4389	64.5967
UGA	24.3610	220.7739	798.5857	26.8675	296.7500	0.0048	0.5295	549.3080	0.6165	137.2290
UKR	69.4730	76.6072	3661.4563	15.8626	283.7390	0.0015	0.4390	569.6410	0.5252	198.9210
ARE	86.7890	137.5743	42,701.4431	60.7550	301.6990	0.0001	0.1018	575.1930	1.1469	216.7460
GBR	83.6520	276.2631	43,070.4984	9.3161	282.8030	0.0032	0.6187	618.2250	1.5383	231.0650
USA	82.4590	35.8932	65,094.7994	5.5864	281.2720	0.0025	0.3772	543.0490	0.9203	200.5650
URY	95.4260	19.7791	17,688.0150	9.6553	290.9350	0.0044	0.6432	511.6710	0.9447	97.6735
UZB	50.4330	76.2228	1784.0098	29.1380	287.4520	0.0007	0.1688	594.1620	1.1283	64.2448
VUT	25.3940	24.6007	3122.9826	7.8357	298.3150	0.0057	0.7847	685.0010	4.1995	119.4330
VEN	88.2400	32.3290	4400.0000	18.3805	298.9910	0.0055	0.6473	523.0590	1.4287	87.0029
VNM	36.6280	311.0978	3425.0893	24.7097	297.8150	0.0050	0.6229	473.6980	0.5059	157.3470
YEM	37.2730	55.2341	750.5546	52.8095	299.5080	0.0004	0.1360	772.2610	1.3657	128.3160
ZMB	44.0720	24.0265	1305.0010	22.5334	295.7210	0.0028	0.5334	718.8830	1.7153	102.2290
ZWE	32.2100	37.8583	1316.7407	16.2415	295.6630	0.0017	0.4246	784.6430	2.0616	94.4050
